# Modulating effect of Xuanfei Baidu granule on host metabolism and gut microbiome in rats

**DOI:** 10.3389/fphar.2022.922642

**Published:** 2022-09-06

**Authors:** Qiaoyu He, Yumeng Shi, Hong Xing, Qian Tang, Jing Liu, Chunxia Li, Han Zhang, Boli Zhang, Junhua Zhang, Xiaopeng Chen

**Affiliations:** State Key Laboratory of Component-Based Chinese Medicine, Tianjin University of Traditional Chinese Medicine, Tianjin, China

**Keywords:** intestinal flora, short-chain fatty acid (SCFA), metabolomics (OMICS), immunity, inflammation, Xuanfei Baidu

## Abstract

Xuanfei Baidu granule (XFBD) is a recommended patented drug for the prevention and treatment of Corona Virus Disease 2019 (COVID-19), which is approved by the National Medical Products Administration. XFBD suppresses the over-activated immune response caused by inflammatory factor storms in COVID-19 infection. The intestine plays a crucial role in the immune system. The mass spectrometry based fecal metabolomics with 16S rDNA sequencing were combined to evaluate the effects of XFBD on host metabolism and gut microbiome. Short-chain fatty acids (SCFAs) contents in fecal matter were quantified by gas chromatography-mass spectrometry (GC-MS). Plasma samples were used to detect immune and inflammatory levels. The results were verified with a rat model of intestinal disorder. Results indicated that XFBD could increase the immune level of Immunoglobulin A (IgA), Immunoglobulin G (IgG) and Immunoglobulin M (IgM) (*p* < 0.05). The OPLS-DA analysis results showed that a total of 271 differential metabolites (178 up-regulated and 93 down-regulated) were identified based on the VIP ≥1, *p* < 0.05, FC ≥ 2 and FC ≤ 0.5. The metabolic pathways mainly involved D-Glutamine and D-glutamate metabolism, Arginine biosynthesis, Biotin metabolism, et al. XFBD modified the gut bacteria structure according to the principal component analysis (PCA), that is, 2 phyla, 3 classes, 5 orders, 11 families and 14 genera were significantly different based on taxonomic assignment. In addition, it could partially callback the relative abundance of intestinal microflora in bacterial disorder rats caused by antibiotics. It is suggested that the intervention mechanism of XFBD might be related to the regulation of intestinal flora composition. The evidence obtained in the study provides a useful reference for understanding the mechanism of XFBD.

## 1 Introduction

Corona Virus Disease 2019 (COVID-19) has spread rapidly since the outbreak at the end of 2019. The early symptoms include fever, cough and fatigue. The strong immune response and cytokine storm in patients with COVID-19 infection are important factors leading to the development of severe diseases (Huang et al., 2020). Meanwhile, the main features of some patients are gastrointestinal symptoms. COVID-19 patients have intestinal flora imbalances, and the changes in microflora are related to the severity of the disease (Tang et al., 2020).

The gut microbiome is a crucial factor for shaping and modulating immune system responses (Schirmer et al., 2016). The mutual interaction between the gut microbiota and the host is further highlighted by its role in sustaining the maturation and functioning of the host’s immune system contributing to its homeostasis (Lavelle and Sokol, 2020). Therefore, the regulation of intestinal flora is an important part of the overall efficacy in the treatment of COVID-19.

Traditional Chinese medicine (TCM) has been proven effective for COVID-19 treatment (Liu et al., 2020). Xuanfei Baidu is one of the 3 preferred prescriptions in the “COVID-19 diagnosis and treatment plan (eighth Edition)” issued by the National Health Commission of the People’s Republic of China and the State Administration of TCM (Zhou et al., 2021). The XFBD decoction is mainly made from Ephedra sinica Stapf [Ephedraceae; Ephedrae Herba] 6 g, Prunus armeniaca L. [Rosaceae; Armeniacae semen amarum]15 g, Gypsum Fibrosum 30 g, Coix lacryma-jobi var. ma-yuen (Rom.Caill.) Stapf [Poaceae; Coicis semen] 30 g, Atractylodes lancea (Thunb.) DC. [Asteraceae; Atractylodis Rhizoma] 10 g, Pogostemon cablin (Blanco) Benth. [Lamiaceae; Pogostemonis Herba] 15 g, Reynoutria japonica Houtt. [Polygonaceae; Polygoni Cuspidati Rhizoma et Radix] 20 g, Verbena officinalis L. [Verbenaceae; Verbenae Herba] 30 g, Phragmites australis (Cav.) Trin. ex Steud. [Poaceae; Phragmitis Rhizoma] 30 g, Descurainia sophia (L.) Webb ex Prantl [Brassicaceae; Descurainiae Semen Lepidii Semen] 15 g, Citrus maxima (Burm.) Merr. [Rutaceae; Citri Crandis Exocarpium] 15 g, Artemisia annua L. [Asteraceae; Artemisiae Annuae Herba] 12 g, Glycyrrhiza glabra L. [Fabaceae; Glycyrrhizae Radix et Rhizoma] 10 g. (The plant names is based on Kew Science https://mpns.science.kew.org/mpns-portal/? and the 2020 version of the Chinese Pharmacopoeia.) To prepare the XFBD extract, XFBD herbs (238 g) were extracted with 400 ml water for 60 min, and extraction was based on COVID-19 diagnosis and treatment plan (Eighth Edition). The XFBD can significantly improve the clinical symptoms of COVID-19 patients, improve immunity and play an anti-inflammatory role (Xiong et al., 2020).

Metabonomics is an important part of systems biology. It is a strong tool to explore the regulation mechanism of metabolism under drug action or disease state from the overall level of metabolites and provides a new possibility for disease prevention and treatment. Meanwhile, it provides a new method for studying the mechanism of the multi-target effect of traditional Chinese medicine compounds (Nicholson et al., 1999; Wu et al., 2019).

This study is based on UHPLC-Q-TOF/MS fecal metabolomics combined with a 16S rDNA sequencing approach to study the effects of XFBD on the fecal metabolic spectrum and intestinal flora composition of normal rats to reveal the regulatory effect of XFBD on the overall metabolism and intestinal flora. At the same time, the results are verified with a rat model of intestinal disorder. It can provide a useful reference for a better understanding of the mechanism of XFBD.

## 2 Materials and methods

### 2.1 Reagents and materials

Xuanfei Baidu granule was provided by Tianjin Modern Innovation Chinese Medicine Technology Co., Ltd. (batch number TRT200329). Acetonitrile, methanol (LC-MS, Fisher, United States); Formic acid (LC-MS, ANRQUA CHEMICALS, United States); Wastons drinking water (Watsons, Guangzhou, China); Acetic acid (AA), Propionic acid (PA), Isobutyric acid (IBA), Butyric acid (BA), Valeric acid (VA), Hexanoic acid (HA), Isovaleric acid (IVA), 2-methyl valeric acid, Methyl tert-butyl ether (CNW Technologies, Germany) (aladdin, China); Phosphoric acid (Shanghaihushi, China), Hastatoside (batch No: DSTDJ006301), Verbenalin (batch No: DSTDM007201), Polydatin (batch No: DSTDH003801), Acteoside (batch No: wkq20042004), Naringin (batch No: 150913) and Glycyrrhizic acid (batch No: wkq20022509). Reference standards including Hastatoside, Verbenalin, Polydatin were bought from Chengdu Desite Bio-Technology Co., Ltd. (Chengdu, China), Acteoside and Glycyrrhizic acid were bought from Sichuan Weikeqi Bio-Technology Co., Ltd. (Sichuan, China), Naringin was bought from National Institutes for Food and Drug Control (Beijing, China). IgG (batch No: CSB-E07981r), IgM (batch No: CSB-E07978r), IgA (batch No: CSB-E07987r) and TNF-α (batch No: CSB-E11987r) levels were determined using a commercially available ELISA kit (Wuhan Huamei Biological Engineering Co., Ltd., Wuhan, China).

### 2.2 Composition analysis of XFBD

A Shimadzu UHPLC system equipped with a photodiode array detector (PDA) was used to qualitatively and quantitatively analyze multiple components in XFBD granule refer to the published article (Wang et al., 2021). Briefly, 2 g of XFBD was extracted with ultrapure water (1:25, g/ml) in an ultrasonic water bath for 30 min. The solution was diluted with 50% methanol at the ratio of 1:1 and vortex-mixed for 5 min. And then centrifuged at 18,407 g for 10 min. The supernatant was filtered through a 0.22 μm filter membrane. An aliquot (2 μL) of the supernatant solution was injected into UHPLC-PDA for analysis. In detail, chromatographic separations were performed on a Shim-pask GISS C18 column (2.1 mm × 100 mm, 1.9 μm, Shimadzu, Japan) using solvent A (0.1% formic acid aqueous solution) and B (acetonitrile) as the mobile phase for gradient elution. The flow rate was 0.3 ml/min. The column temperature was 40°C and the injection volume was 2 μl. PDA detection wavelength was 254 nm. The gradient elution was carried out at 0–8 min, 5%–10% B; 8–13 min, 10%–15% B; 13–18 min, 15%–17% B; 18–30 min, 17%–45% B; 30–35 min, 45%–95% B.

### 2.3 Animal treatment and sample collection

Thirty-two male Sprague-Dawley (SD) rats (weighing approximately 180–200 g) were supplied by Beijing Huafukang Biotechnology Co., Ltd. (licenses approval number: SCXK (Jing) 2019-0008). All the rats were kept in a laboratory with controlled temperature and humidity (room temperature: (25 ± 2)°C, humidity: (50 ± 15)%) and a 12-h light/dark cycle and free access to food and water. All experimental protocols strictly followed the Guide for the Care and Use of Laboratory Animals (NIH publication 85-23, revised 1985), the Guidance Suggestions for the Care and Use of Laboratory Animals issued by the Ministry of Science and Technology of China (2006), and the Animal Use and Care Committee of Tianjin University of Traditional Chinese Medicine (No. IRM-DWLL-2019033).

The normal rats: The rats adapted to the living environment for 1 week and were randomly divided into two groups: the control group (eight rats) and the XFBD group (eight rats). The rats in the XFBD group were fed with XFBD by daily oral gavage, and the dose was determined as 2.5 g·kg^−1^·d^−1^ according to the 6.25 times of the converted surface area based on the clinical dose 20 g·d^−1^. The rats in the control group were administered with the same volume of sterilized saline. After a 4-weeks experimental period, all rats were sacrificed. After 10% chloral hydrate anesthesia, the blood samples of the abdominal aorta of rats in each group were taken in an anticoagulant tube, and then centrifuged for 10 min at 4°C 4,602 g. The supernatant was collected into the EP tube and stored at −80°C. The fecal matter was collected in metabolic cages and kept at −80°C for further use. The plasma samples of three rats in each group were randomly selected to detect the plasma biochemical indexes. Fecal samples were selected for 16S rDNA gene sequencing, SCFAs content determination, and metabolomics analysis.

Bacterial disorder rats: 16 male SD rats (180–220 g) were adaptively fed for 1 week, and the antibiotics (imipenem/cilastatin 50 mg·kg^−1^·d^−1^) were given by oral gavage. After 4 days of antibiotics, the rats were randomly divided into the spontaneous recovery group (feeding saline, eight rats) and the XFBD group (feeding Xuanfei Baidu granule, eight rats), the dosage of XFBD was 2.5 g·kg^−1^·d^−1^. Fecal samples were collected after 10 days and stored in a refrigerator at −80°C for 16S rDNA gene sequencing.

### 2.4 Detection of plasma biochemical indexes

The plasma from each group was used to detect the level of IgG, IgM, IgM and TNF-α, and the test was carried out in strict accordance with the instructions of the kit.

### 2.5 Sample preparation

The treatment process of feces samples used for fecal metabolomics analysis was as follows: feces samples were taken out at −80°C and placed in a freeze dryer for drying. A total of 50 mg feces from each sample was placed in a 2 ml sterile tube. After screening and optimization, 900 μl precooled acetonitrile and 300 μl precooled distilled water that acted as extraction solvent was added to each sample, which contained 2 μg·mL^−1^ leucine enkephalin as the internal standards. Then, a steel ball having a particle diameter of 5 mm and a particle diameter of 3 mm was added to the sterile tube and used the tissue homogenizer (Shanghai Jingxin Industrial Development Co., Ltd., Tissuelyser-32, China) to homogenize at 60 Hz for 120 s. Then, the mixture was treated with ultrasound for 5 min on an ice bath. After blending and centrifuging at 21,130 g for 15 min at 4°C, the supernatant was obtained. Then, samples were filtered through a 0.22 μm microporous membrane for UHPLC-Q-TOF/MS analysis. To ensure the stability of the entire analysis system, quality control samples (QC) were used for method verification. Ten microlitres of each prepared sample extraction was randomly selected from each group and mixed as the QC samples, and for every 10 analytical samples, one QC sample was used.

Levels of short-chain fatty acids (SCFAs) were determined by direct measurements of fecal samples and the preparation method was as follows: The fecal samples of 20 mg were accurately weighed and placed in a 2 ml EP tube. Then add 1 ml of phosphoric acid (0.5% v/v) solution and a small steel ball into the EP tube. The mixture was grinded for 10 s, three times, then vortexed for 10 min and ultrasonicated for 5 min. After centrifugation at 4°C and 13,523 g for 10 min, the supernatant of 0.1 ml was added to a 1.5 ml centrifugal tube. Add 0.5 ml methyl tert-butyl ether (MTBE) (containing internal standard) solution to the centrifugal tube. The mixture was vortexed for 3 min and ultrasonicated for 5 min. After that, the mixture was centrifuged at 13523 g for 10 min at the temperature of 4°C. The supernatant was collected and used for GC-MS/MS analysis.

### 2.6 Instrumentation and conditions

Fecal metabolomics data acquired by LCMS-9030 Ultra High Performance Liquid Chromatography Quad-rupole Time-of-Flight Mass Spectrometer (Shimadzu, Japan). The chromatography separation condition in UHPLC-Q-TOF/MS was performed on a Shim-pask GISS C18 column (2.1 mm × 100 mm, 1.9 μm) (Shimadzu, Japan) at 35°C. The mobile phase for fecal samples was composed of 0.1% formic acid in water (phase A) and acetonitrile (phase B). Gradient elution was used and set as follows: 0–7 min, 5%–45% B; 7–14 min, 45%–95% B; 14%–15.5 min, 95% B; 15.5–16 min, 95%–5% B; 16–20 min, 5%–5% B. The injection volume was 2 μl, and the flow speed was 0.2 ml/min.

MS operation conditions were as follows: The Q-TOF/MS spectrometer was configured with an electrospray ion source (ESI) operating in the negative and positive ion modes. Ion source interface voltage: 3.0 kV. Nitrogen was used as the drying gas and the atomizing gas. The drying gas flow rate was set to 10 L/min, and the atomizing gas flow rate was set to 3.0 L/min. Used air as heating gas and set the flow rate to 10 L/min; used argon as collision gas; desolvent tube temperature was 250°C; heating block temperature was 400°C; interface temperature was 300°C; Scan mode: MS Scan (m/z 80–560; 550–1,000), MS2 (m/z 50–560; 50–1,000); The collision energy (CE) was 35 ± 17V.

Agilent 7890B gas chromatograph coupled to a 7000D mass spectrometer with a DB-5MS column (30 m length × 0.25 mm i.d. × 0.25 μm film thickness, J&W Scientific, United States) was employed for GC-MS/MS analysis of fecal samples. Helium was used as the carrier gas, at a flow rate of 1.2 ml/min. Injections were made in the splitless mode and the injection volume was 2 μl. The oven temperature was held at 90°C for 1 min, raised to 100°C at a rate of 25°C/min, raised to 150°C at a rate of 20°C/min, hold for 0.6 min, raised to 200°C at a rate of 25°C/min, hold on 0.5 min, after running for 3 min. All samples were analyzed in multiple reaction monitoring mode. The injector inlet and transfer line temperature were 200°C and 230°C, respectively (Bianchi et al., 2011; Zhao et al., 2017).

### 2.7 Metabolites identification and pathway enrichment analysis

Data format conversion: All the obtained mass spectrometer off-machine data were converted into data files in mzML format by the file format converter that comes with the Labsolutions software (Shimadzu, Japan). XCMS program was used for peak extraction, alignment and retention time correction. The peak area was corrected by “SVR” method, and the peaks with a missing rate of more than 50% in each group were filtered. The screened peaks were corrected, and the metabolites identification information was obtained by searching the laboratory self-built database and integrating the public database including HMDB, Metlin and MetDNA methods. Finally, statistical analysis was carried out by the R program. The variables which met screening conditions (VIP ≥1, *p* < 0.05, FC ≥ 2 and FC ≤ 0.5) were deemed to be potential metabolite bio-markers. Finally, MetaboAnalyst 5.0 software (http://www.metaboanalyst.ca/) was used for metabolic pathway enrichment analysis.

### 2.8 16S rDNA microbial community analysis

Total genome DNA from samples was extracted using Cetyltrimethylammonium Bromide method (CTAB) (Griffith et al., 2009; Greathouse et al., 2019). DNA concentration and purity were monitored on 1% agarose gels. According to the concentration, DNA was diluted to 1 ng/μl using sterile water. V3-V4 hypervariable region of 16S DNA genes was targeted and amplified by using specific primers with 515F (5′-ACT​CCT​ACG​GGA​GGC​AGC​A-3′) and 806R (5′-GGACTACHVGGGTWTCTAAT-3′). Mix the same volume of 1X loading buffer (contained SYB green) with PCR products and operate electrophoresis on 2% agarose gel for detection. PCR products were mixed in equidensity ratios. Then, the mixture PCR products were purified with Qiagen Gel Extraction Kit (Qiagen, Germany). NovaSeq PE250 platform (Illumina Inc., San Diego, CA) was used for sequencing.

### 2.9 16S rDNA sequencing and data analysis

Data split: Paired-end reads was assigned to samples based on their unique barcode and truncated by cutting off the barcode and primer sequence. Sequence assembly: Paired-end reads were merged using FLASH (V1.2.11, http://ccb.jhu.edu/software/FLASH/) (Magoč and Salzberg, 2011), a very fast and accurate analysis tool, which was designed to merge paired-end reads when at least some of the reads overlap the read generated from the opposite end of the same DNA fragment, and the splicing sequences were called raw tags. Data Filtration: Quality filtering on the raw tags was performed under specific filtering conditions to obtain the high-quality clean tags according to the QIIME(V1.9.1, http://qiime.org/scripts/split_libraries_fastq.html) quality controlled process. Chimera removal: The tags were compared with the reference database (Silva database, http://www.arb-silva.de/) using UCHIME algorithm (UCHIME, http://www.drive5.com/usearch/manual/uchime_algo.html) to detect chimera sequences, and then the chimera sequences were removed. Then the Effective Tags were finally obtained (Haas et al., 2011). Then, sequence analyses were performed using Uparse software (Uparse v7.0.1001, http://drive5.com/uparse/). According to 97% similarity, it was considered to be the high-quality operational taxonomic units (OUT) (Edgar, 2013). For each representative sequence, the Silva Database (http://www.arb-silva.de/) was used based on Mothur algorithm to annotate taxonomic information. To study the phylogenetic relationship of different OTUs, and the difference of the dominant species in different samples (groups), multiple sequence alignment was conducted using the MUSCLE software (Version 3.8.31, http://www.drive5.com/muscle/). PICRUSt2 contains an updated database of gene families and reference genomes. It also provides interoperability with any operational taxonomic unit (OTU)-picking or denoising algorithm and enables phenotype predictions. According to the KEGG database, the metabolic functions of bacterial communities were predicted using the PICRUSt2 and annotated to their biological function.

### 2.10 Correlation analysis between gut microbiota and fecal metabolites

Correlation coefficients between SCFAs and changes in bacterial genus relative abundance were calculated using Spearman’s correlation analysis test. Spearman’s correlation coefficient was used to express the relationship between various parameters, the values range from −1 to 1. Positive numbers indicate a positive correlation, negative numbers indicate a negative correlation, and the greater the absolute value, the greater the correlation. The correlation analysis was calculated using the cor function of R software.

### 2.11 Statistical analysis

SPSS 22.0 version software and Origin 2019b were used to analyze the serum biochemical indicators, the unpaired two-tailed Student’s t-test was used between the two groups. The experimental results were expressed in the form of mean ± standard deviation (SD), and a value of *p* < 0.05 indicated statistical significance. In 16S rDNA analysis, Lefse analysis was used to screen out marker species with significant differences between groups, the statistical analysis was carried out by R software.

## 3 Results

### 3.1 Composition analysis of XFBD

The chromatograms of the mixed six standards and XFBD extracted solution were shown in Supplementary Figure S1. The results showed that the contents of hastatoside, verbenalin, polydatin, acteoside, naringin and glycyrrhizic acid in the XFBD extract were 4.93, 4.03, 5.38, 1.86, 28.68, and 2.83 mg/g, respectively.

### 3.2 Effect on immunoregulation and cytokine production

**FIGURE 1:**
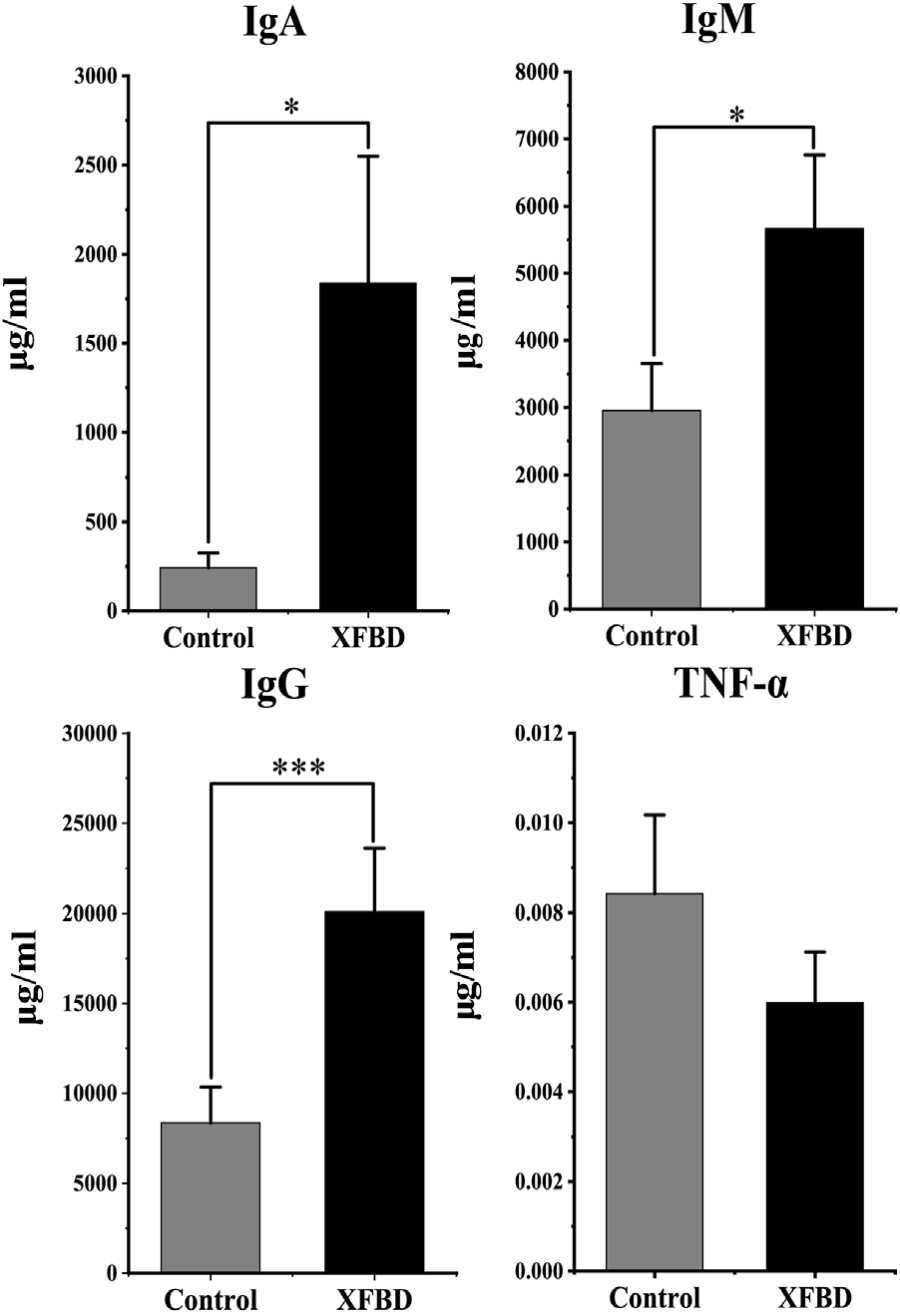
Changes in immunoglobulin A (IgA), IgM, IgG and TNF-α levels between control and XFBD groups.

The humoral immune response after XFBD intervention was evaluated by determining IgA, IgG, and IgM in the plasma while inflammation by TNF-α. As shown in Figure 1, compared with the control group, the levels of IgA, IgG, and IgM increased significantly after intervention with XFBD (*p* < 0.05). The TNF-α had no statistical differences (*p* > 0.05). The results showed that XFBD can regulate the expression of immune factors *in vivo*.

### 3.3 Effect of XFBD intervention on fecal metabolomic profiles

#### 3.3.1 Fecal metabolomics analysis

**FIGURE 2:**
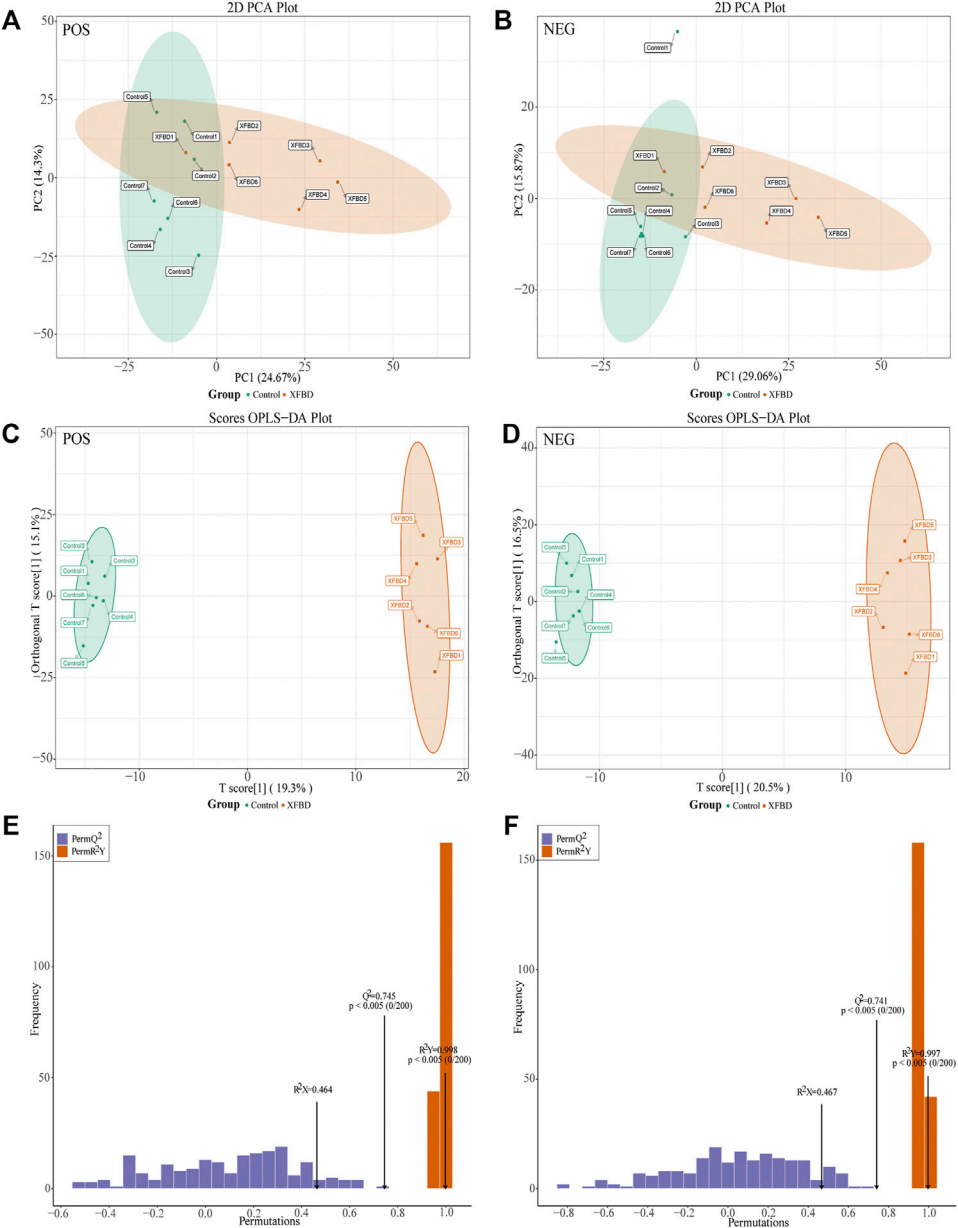
Metabolic profiling between control and XFBD group. **(A)**Score plot of PCA model in positive ion mode. **(B)** Score plot of PCA model in negative ion mode. **(C)** Scores plot of the OPLS-DA model in positive ion mode. **(D)** Scores plot of the OPLS-DA model in negative ion mode. **(E)** The plot of the permutation test (200 times) of the OPLS-DA model in positive ion mode. **(F)** The plot of the permutation test (200 times) of the OPLS-DA model in negative ion mode.

All the tested samples were discriminated in the PCA and OPLS-DA models. As shown in Figures 2A–D, a significant separation was observed between the control group and the XFBD group, which indicated that the composition of fecal metabolites changed significantly after the intervention of XFBD. To characterize whether the OPLS-DA model was overfitted, 200 detection groups were randomly selected for prediction with this model. As shown in Figures 2E,F, the results showed that the model was not overfitting, the established statistical model had great discrimination, adaptability and predictive ability.

Using laboratory self-built databases, online HMDB and Metlin databases, and metDNA methods for screening, a total of 271 potential biomarkers were screened, including 178 up-regulated metabolites and 93 down-regulated metabolites (Supplementary Table S1). To show the difference in the expression of metabolites, a cluster analysis was performed on the top 30 metabolites ranked by VIP value (Supplementary Figure S2). The abscissa represents the sample name, and the ordinate represents the top 30 differential metabolites according to the VIP value. In the figure, the red color represents the up-regulation of metabolites, and the green color represents the down-regulation of metabolites. The results showed that there were significant differences in metabolite levels between groups.

#### 3.3.2 Metabolic pathway analysis

**FIGURE 3:**
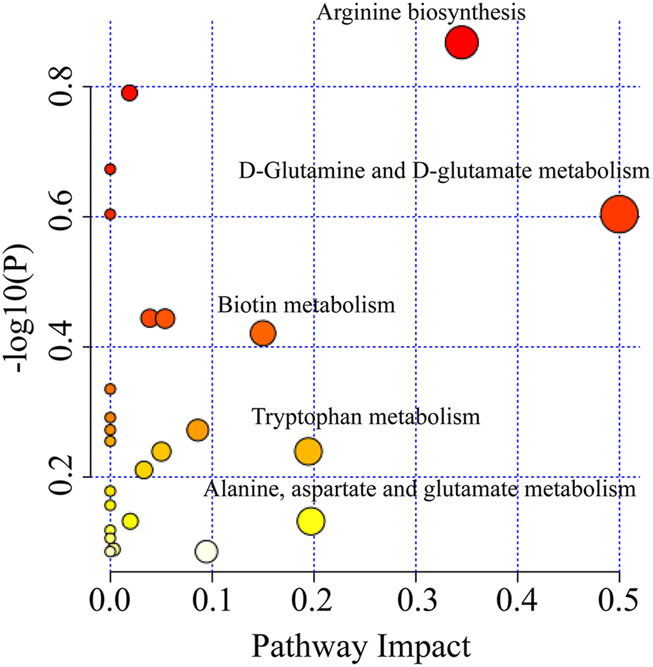
Enrichment of pathway analysis with MetaboAnalyst.

The metabolic pathways of 271 different metabolites were analyzed to further find out the metabolic pathways regulated by XFBD. Metabolic pathways were considered significantly relevant with XFBD intervention when the impact value was greater than 0.1. These pathways mainly involved D-Glutamine and D-glutamate metabolism, Arginine biosynthesis, Biotin metabolism, Tryptophan metabolism, Alanine, aspartate and glutamate metabolism (Figure 3).

### 3.4 Effect of XFBD intervention on gut microbiota composition in the normal rats

#### 3.4.1 OTU classification statistics

In total, 1088333 V3-V4 16S rDNA high-quality sequences were detected from 16 fecal samples. After quality filtering, 33328 operational taxonomic units (OTUs) were determined (at 97% similarity cut-off). Principle coordinates (PCoA) analysis using unweighted Unifrac distance was applied to assess the relationships between samples and groups. The results showed distinct clustering of gut microbiota composition in the control and XFBD groups (Supplementary Figure S3). Venn diagrams presented that the distribution of OTUs was shared and unique between the two groups (Supplementary Figure S4). To further analyze the differences in the composition of intestinal microbial community among two groups, the structural distribution of intestinal microflora among different groups was compared based on non-metric multidimensional scaling (NMDS). NMDS analysis was not affected by the value of sample distance, and the difference in intestinal flora composition among different groups was verified by ranking the sample distance. It was generally believed that when the Stress of NMDS was less than 0.2, the result of NMDS analysis was more reliable (Supplementary Figure S5). The results showed that there was an obvious trend of separation between the two groups and the Stress was 0.17. Compared with the control group, the composition of intestinal flora in the XFBD group changed significantly.

#### 3.4.2 XFBD regulates the gut microbiota diversity and composition of the gut microbiota in rats

Alpha diversity is mainly used to evaluate the diversity, richness and uniformity of microbial communities, evaluated by species richness index (Chao1, observed species) (Chao, 1984) and bacterial diversity index (Shannon, Simpson) (Simpson, 1949; Shannon, 2001). The rarefaction curve drawn based on OTUs per sample was flat, suggesting a reasonable sequencing depth (Supplementary Figure S6).

**FIGURE 4:**
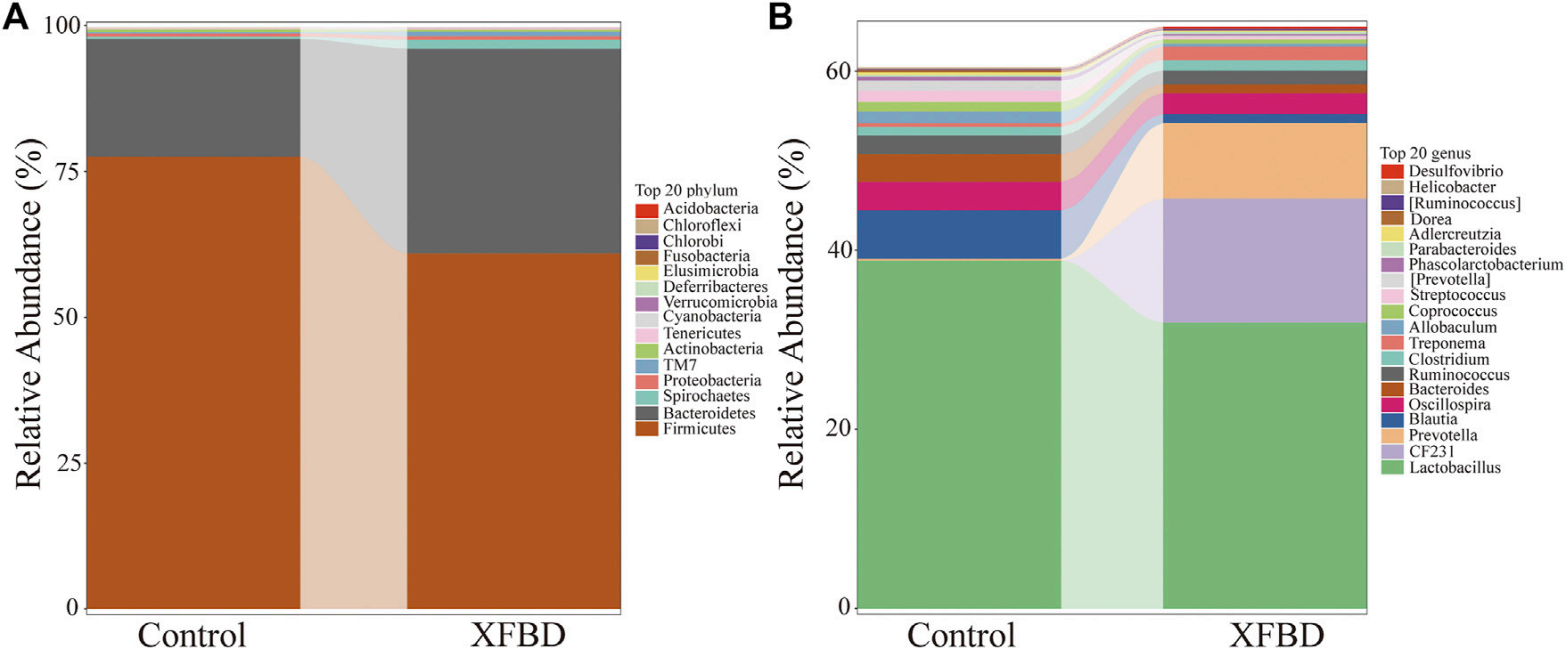
Differences of gut microbiota at the phylum and genus level **(A)** Stacking diagram of species distribution at the phylum level. **(B)** Stacking diagram of species distribution at the genus level.

The effects of XFBD were analyzed at phylum and genus levels. The t-test was used to analyze the significance of the groups. Gut microbiota at the phylum level consisted mostly of *Firmicutes*, *Bacteroidetes*, *Spirochaetes*, *Proteobacteria*. *Firmicutes* and *Bacteroidetes* represented more than 95% relative abundance. As shown in Figure 4A, there were differences between these two groups. Compared with the control group, the main intestinal flora changed after XFBD intervention, in which the relative abundance of *Firmicutes* decreased from 77.5% to 60.9%. The relative abundance of *Bacteroides* and *Spirochaetes* increased from 20.2% to 35.1% and 0.38%–1.54% respectively. Compared with the control group, the relative abundance of *Fusobacteria* in the XFBD group was significantly lower than that in the control group (*p* < 0.05) while *TM7* increased significantly (*p* < 0.01). To further understand the detailed alternations of gut microbiota, the relative abundance changes in bacteria’s genus levels were analyzed. The relative abundance histogram of the species with the top 20 abundance at the genus level for each sample was shown in Figure 4B. In terms of species abundance, there was a great difference in species abundance between the control group and the XFBD group. Compared with the control group, the abundance of *Prevotella*, *Desulfovibrio*, *Paraprevotella*, *Odoribacter*, *Paenisporosarcina*, *CF231*, *YRC22* and *Butyricimonas* increased significantly in the XFBD group, which may be related to the improvement of colonic mucosal permeability. However, the abundance of *Blautia*, *Allobaculum*, *Phascolarctobacterium*, *Dorea*, *Bifidobacterium* and *Collinsella* decreased significantly (*p* < 0.05).

The relative abundance of the flora at the phylum and genus levels of the control group and XFBD group were compared (Figures 4A,B). The results suggested that XFBD has a regulatory role in balancing probiotics and pathogenic bacteria in gut microbiota. Overall, these changes caused by the XFBD intervention led to an improvement in gut health. *Prevotella* is a gram-negative bacteria and can grow under anaerobic conditions. In this study, *Prevotella* and *Paraprevotella* were up-regulated after XFBD intervention, and there were definite reports that these genera were beneficial to the organism. The research shows that *Prevotella* plays a role in the BKB-induced improvement in glucose metabolism observed in certain individuals, potentially by promoting increased glycogen storage. In addition, increased levels of *Prevotella* may be favored to maintain glucose homeostasis (Zhang et al., 2012; Kovatcheva-Datchary et al., 2015). *Paraprevotella* is a kind of gram-negative anaerobes. Its final product succinic acid can promote the production of butyrate in the intestinal tract and achieve the purpose of anti-inflammation (Morotomi et al., 2009; Borton et al., 2017).

**FIGURE 5:**
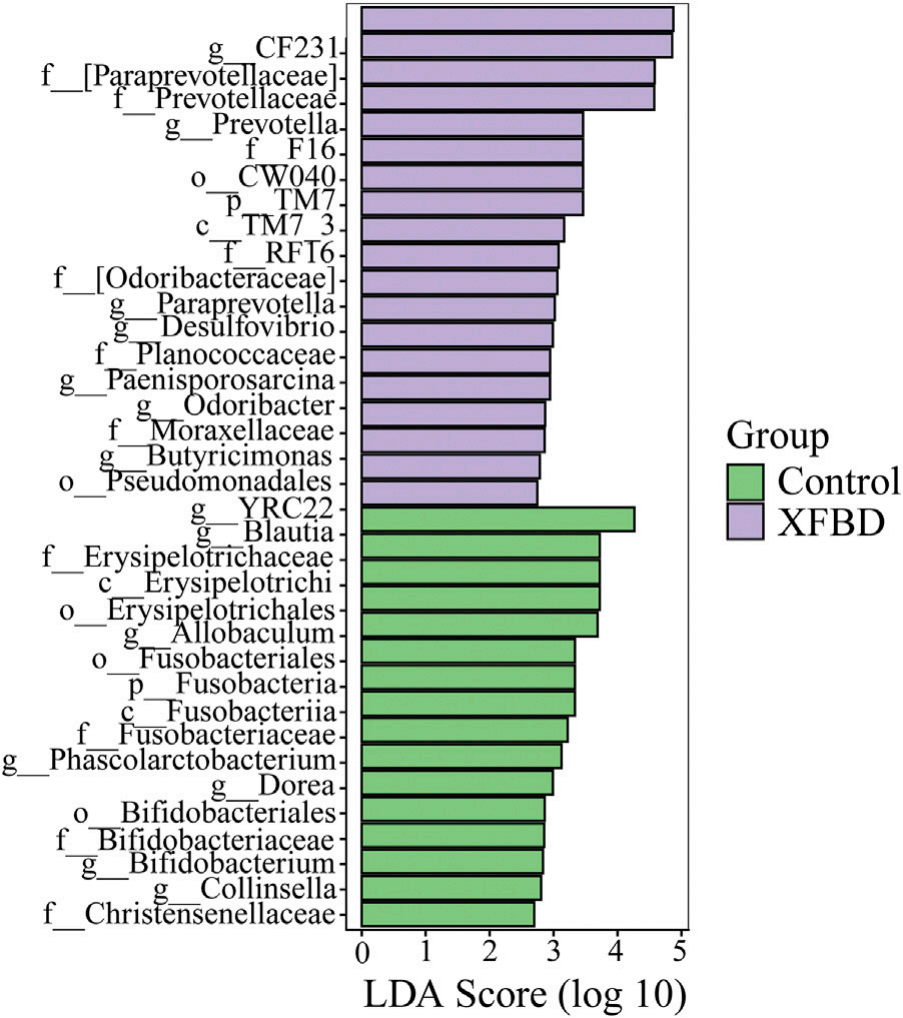
LEfSe analysis of differential abundant species as biomarkers between control and XFBD.

To find the potential microflora with differences between different groups, LDA Effect Size (LEfSe) analysis was used to analyze the differences at all classification levels to find the iconic microflora between groups. LEfSe analysis is an analysis method that combines nonparametric Kruskal–-Wallis and Wilcoxon rank sum test with linear discriminant analysis effect size. It can directly analyze the differences of all taxonomic levels at the same time, and it puts more emphasis on finding robust differences between groups (Segata et al., 2011). The Cladogram was used to show the taxonomic hierarchical distribution of marker species in each group of samples. The results showed that there were 35 taxa with significant differences between the control group and XFBD group, including 2 phyla, 3 classes, 5 orders, 11 families, and 14 genera, as shown in Supplementary Figure S7. The histogram of LDA score showed significant differences among species at different classification levels, and the screening conditions were that LDA was greater than 2 and *p* less than 0.05, as shown in Figure 5. The results of the analysis showed that compared with the XFBD group, 6 genera in the Control group were significantly down-regulated, sorted by LDA from highest to lowest were *Blautia*, *Phascolarctobacterium*, *Dorea*, *Bifidobacterium*, and *Collinsella*. Compared with the Control group, 8 genera in the XFBD group were significantly up-regulated, sorted by LDA from highest to lowest were *CF231*, *Prevotella*, *Paraprevotella*, *Desulfovibrio*, *Paenisporosarcina*, *Odoribacter*, *Butyricimonas* and *YRC22*.

#### 3.4.3 Function prediction for gut microbiota

**FIGURE 6:**
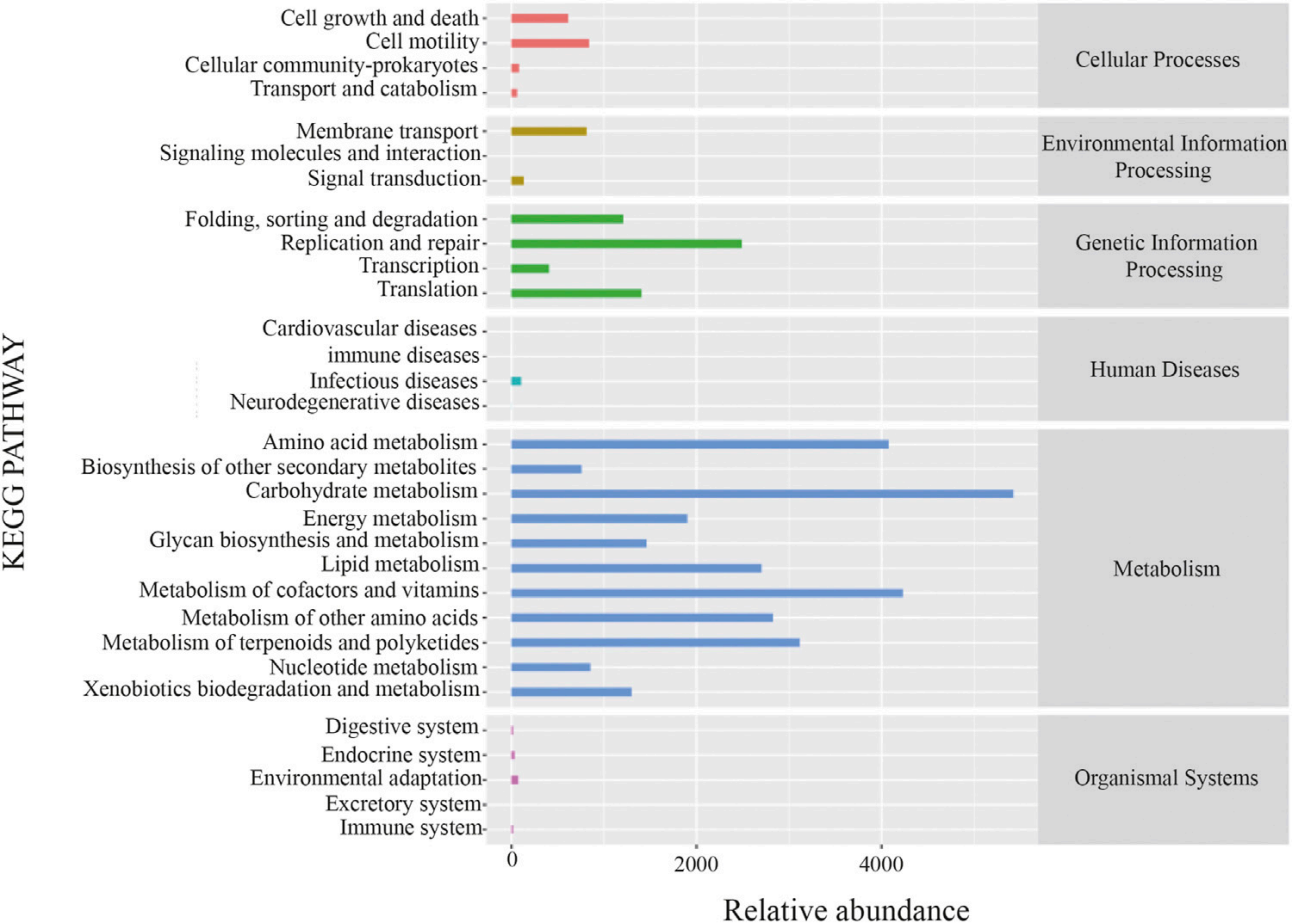
PICRUSt analysis result.

The potential metabolic function of gut microbiota influenced by XFBD was further predicted using the Phylogenetic Investigation of Communities by Reconstruction of Unobserved States (PICRUSt2) analysis. According to the functional prediction map of the metabolic pathway, it was found that KEGG metabolic pathways involved Carbohydrate metabolism, metabolism of cofactors and vitamins, amino acid metabolism and metabolism of terpenoids and polyketides, etc (Figure 6).

### 3.5 Short-chain fatty acids analysis

**FIGURE 7:**
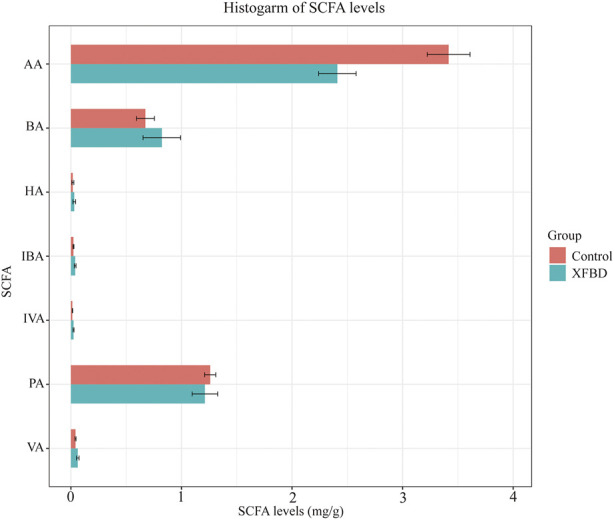
Diagrams of changes in expression levels of short-chain fatty acids. AA, Acetic acid; PA, Propionic acid; IBA, Isobutyric acid; BA, Butyric acid; IVA, Isovaleric acid; VA, Valeric acid; HA, Hexanoic acid.

Short-chain fatty acids, as the most abundant metabolites in the intestinal microbial community, play a vital role in regulating the host immune system and improving inflammatory response. We specifically tested the level of SCFAs in the fecal content of rats, including Acetic acid, Propionic acid, Isobutyric acid, Butyric acid, Isovaleric acid, Valeric acid, Hexanoic acid, as presented in Figure 7. Compared with the control group, the content of acetic acid and propionic acid in the XFBD group decreased, while the contents of the other five kinds of SCFAs increased. Acetic acid changed significantly after the intervention of XFBD (*p* < 0.01).

### 3.6 Spearman’s correlation analysis between SCFAs and gut microbiota

**FIGURE 8:**
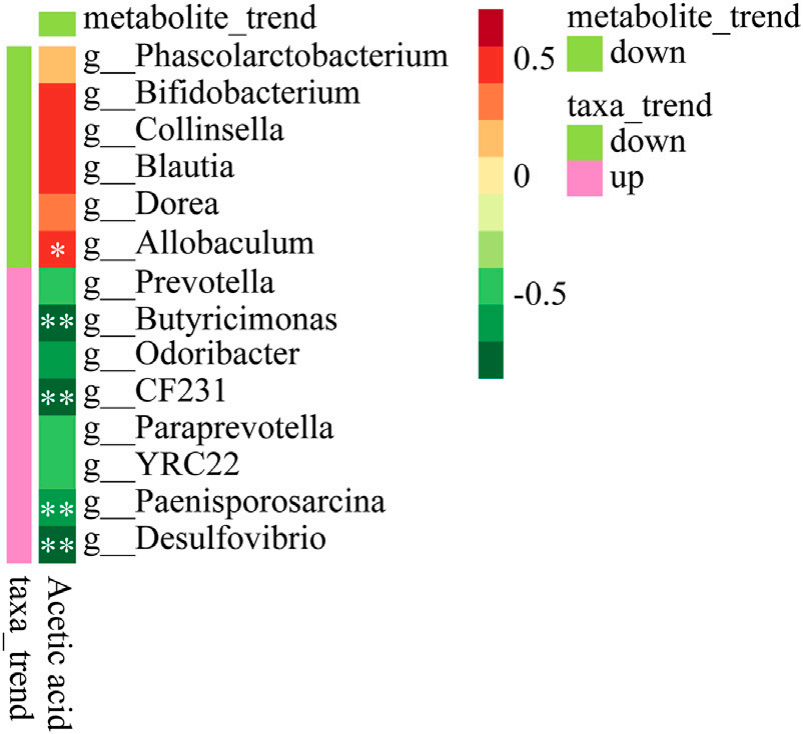
Heat map to show Spearman’s correlation between Acetic acid and microbiota composition at the genus level.

To evaluate whether the SCFAs measured in the feces were associated with the altered gut microbiota, Spearman’s correlation analysis was performed. The correlation heat map showed the correlation between differential microorganisms and differential metabolites at different classification levels. It could be seen from Figure 8 that acetic acid had a significant positive correlation with *Allobaculum* (*p* < 0.05). It had a significant negative correlation with *Butyricimonas*, *CF231*, *Paenisporosarcina*, *Desulfovibrio* (*p* < 0.01). It was suggested that XFBD could play a role by regulating these gut microbiota and further affecting their metabolites.

### 3.7 Correlation analysis between fecal metabolites and gut microbiota

**FIGURE 9:**
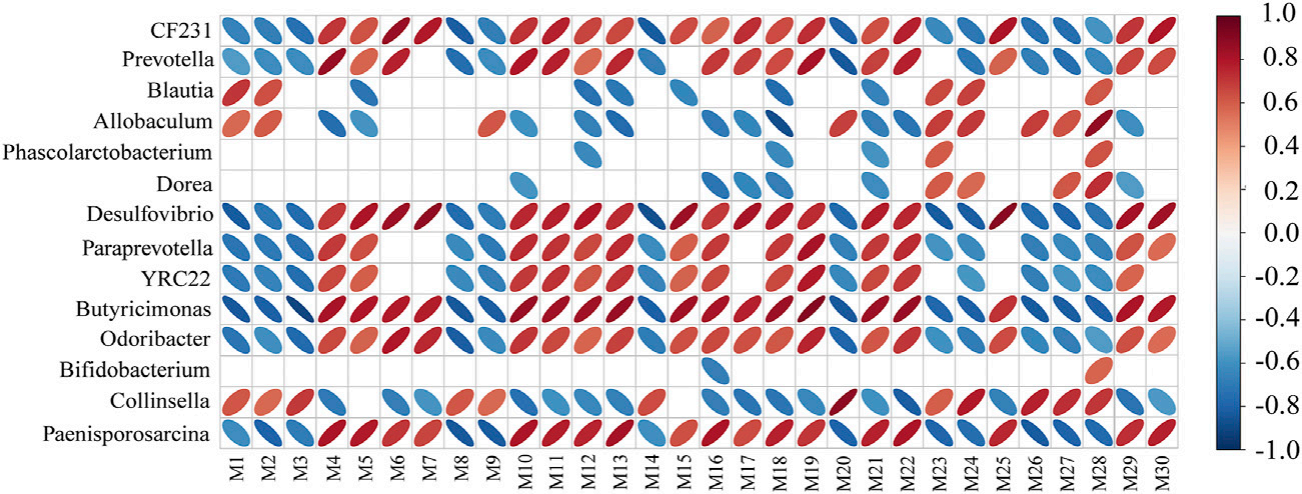
Corrections between the top 30 metabolites ranked by VIP value and intestinal flora. The red ellipse represents a positive correlation, and the blue ellipse represents a negative correlation. The greater the absolute value of the correlation, the thinner the ellipse. A blank grid represents a significant *p* value greater than 0.05.

Spearman’s correlation analysis was also conducted to analyze the correlations between the 14 significantly different genera and the top 30 metabolites ranked by VIP value (Figure 9). The numbers and corresponding metabolites ranked at the top30 according to the VIP value were shown in Table 1. The results showed that there was a strong correlation between 14 different flora and top30 metabolites. It was suggested that the level of metabolites in the body can reflect the structure of gut microbiota.

**TABLE 1:** Top30 metabolites according to VIP value.

No.	Metabolite name
M1	5,7,4′-Trimethoxyflavone
M2	Indole-3-Carboxaldehyde
M3	11,14,17-Eicosatrienoic acid
M4	Chenodeoxycholic acid
M5	Hydrocortisone
M6	MG(18:2(9Z,12Z)/0:0/0:0)
M7	Oleic acid ethyl ester
M8	Ricinoleic acid methyl ester
M9	Coumarin
M10	N-cis-Tetradec-9-enoyl-L-homoserine lactone
M11	Trichlorfon
M12	3beta-Hydroxy-5-cholestenoate;3beta-Hydroxycholest-5-en-27-oic acid
M13	Glu-Asp-Thr
M14	Ionomycin
M15	Ser-Val-Lys-Arg
M16	3-Methyl-5-pentyl-2-furanpentadecanoic acid
M17	7(14)-Bisabolene-2,3,10,11-tetrol
M18	fluticasone 17beta-carboxylic acid
M19	Limaprost
M20	O-Decanoyl-L-carnitine
M21	Phenylalanyl-Isoleucine
M22	1-Naphthol β-D-glucuronide
M23	Rifaximin
M24	Asp-Ile-Ser-Glu
M25	Met-Thr-OH
M26	TyrMe-Tyr-OH
M27	10E,12Z-Octadecadienoic acid
M28	3,5-Dihydroxybenzoic acid
M29	(3beta,17alpha,23S)-17,23-Epoxy-3,29-dihydroxy-27-norlanost-8-en-24-one
M30	10′-Apo-beta-carotenal

### 3.8 XFBD regulates the composition of intestinal microbiota in microbiome disorder rats

**FIGURE 10:**
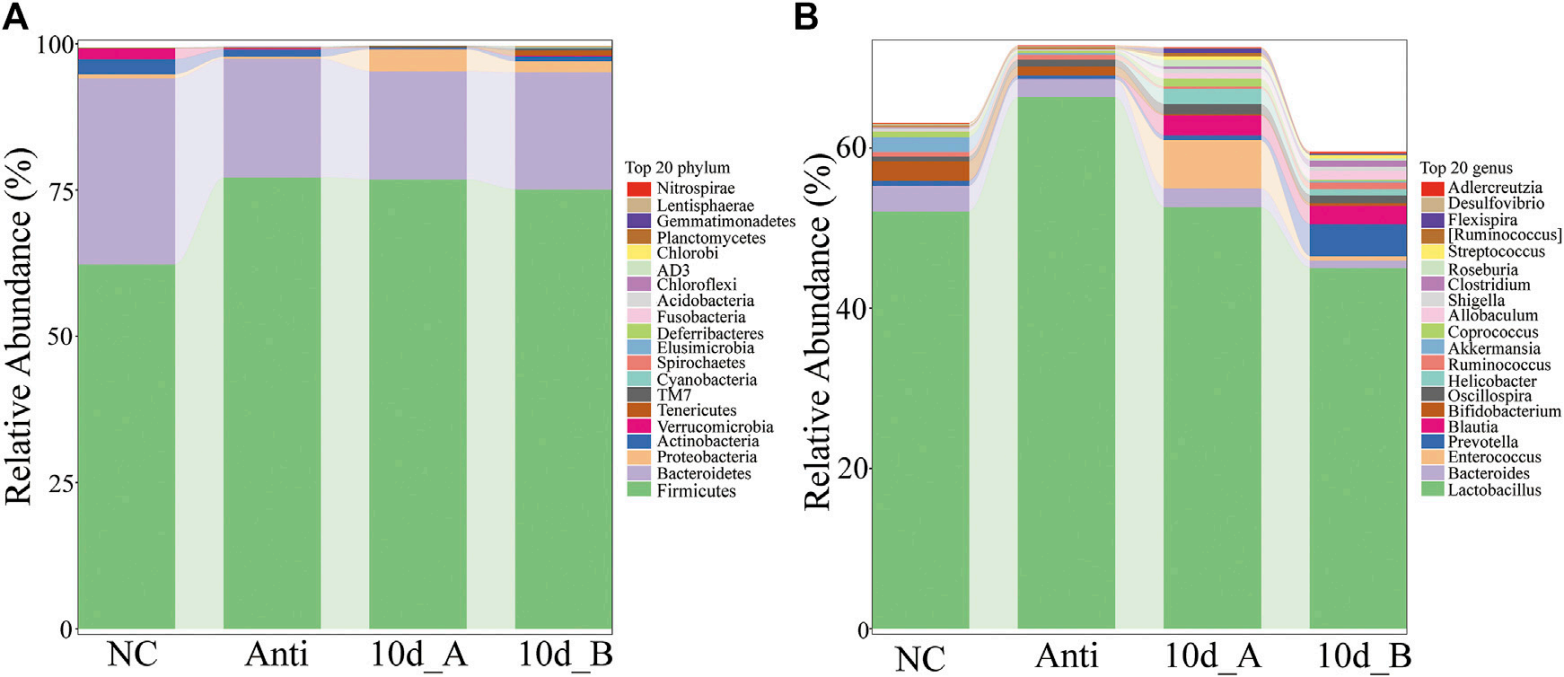
Differences of intestinal flora at the level of phylum and genus in rats with bacterial flora disorder. **(A)** Stacking diagram of species distribution at the phylum level (the normal group: NC; Antibiotic group: Anti; the spontaneous recovery group: 10d_A; XFBD group: 10d_B). **(B)** Stacking diagram of species distribution at the genus level (the normal group: NC; Antibiotic group: Anti; the spontaneous recovery group: 10d_A; XFBD group: 10d_B).

In this study, we further investigated the effect of XFBD on the composition of intestinal flora in rats with Intestinal bacterial disorder. The results indicated that XFBD has a callback effect on the disturbance of intestinal flora induced by antibiotics in rats. At the phylum level, compared with the spontaneous recovery group, the relative abundances of *Firmicutes* and *Proteobacteria* in the XFBD group were decreased, while *Bacteroidetes*, *Actinobacteria*, *Verrucomicrobia* and *Deferribacteres* were increased, and gradually tended to the normal group. There was a significant difference in the relative abundance of *Acidobacteria* between the two groups (*p* < 0.05) (Figure 10A). At the genus level, there were significant differences in 12 genera between the spontaneous recovery group and the XFBD group. Among them, the abundance of *Bacteroides* and *Alistipes* in the XFBD group decreased significantly (*p* < 0.05). However, the relative abundance of *Ruminococcus*, *SMB53*, *Clostridium*, *p-75-a5*, *Corynebacterim*, *Halomonas*, *Collinsella*, *Yonghaparkia*, *Lactococcus* and *Oceanicaulis* increased significantly (*p* < 0.05), and the relative abundance of *Ruminococcus*, *SMB53* and *Yonghaparkia* gradually tended to the normal group (Figure 10B).

## 4 Discussion

COVID-19 is a highly contagious disease caused by infection with SARS-CoV-2. The severity of COVID-19 is mainly related to host factors, especially cellular immune responses in patients (Zhou and Ye, 2021). From the incubation period toward critical illness, the direct cytotoxicity of SARS CoV-2, coagulopathy and intensified immune responses play important roles in the progression to severe diseases (Melenotte et al., 2020). Intestinal flora plays a critical role in maintaining immune homeostasis. The flora can change the structure and function of the immune system, reshape the immune microenvironment, and promote or interfere with the development of specific diseases (Zhou et al., 2020).

In this study, the regulatory effects of XFBD on overall metabolism and intestinal flora in rats were discussed based on metabolomics and intestinal microbiology. The changes in fecal metabolites have been identified by untargeted metabolomics and GC-MS. Meanwhile, the relative abundance of intestinal microorganisms also changed significantly at different classification levels. Intestinal bacterial disorder model results indicate that XFBD had a callback effect on the disturbance of intestinal flora induced by antibiotics in rats. At the phylum level, the relative abundances of *Bacteroidetes*, *Actinobacteria*, *Verrucomicrobia* and *Deferribacteres* were increased and gradually tended to the normal group. At the genus level, the relative abundance of *Ruminococcus*, *SMB53* and *Yonghaparkia* gradually tended to the normal group. These results suggest that the mechanism of action of XFBD may be related to the regulation of the composition of intestinal flora.

XFBD increased the relative abundance of beneficial bacteria such as *Prevotella*, *Paraprevotella*, *Butyricimonas*. Meanwhile, *Paraprevotella* and *Butyricimonas* were newly added species after the intervention of XFBD. *Prevotella* is a genus of *Bacteroides*. Studies have shown that it can degrade polysaccharides and ferment them into short-chain fatty acids (Ou et al., 2013). In addition, it can act as an energy substrate to participate in fiber competition, thus inhibiting the growth of other bacteria (Chen et al., 2017). *Paraprevotella* is a kind of gram-negative anaerobes. Its content in healthy people is higher than in patients with irritable bowel syndrome. It can produce short-chain fatty acids, which can reduce intestinal stress and maintain intestinal homeostasis (Valdez-Palomares et al., 2021; Zhang et al., 2021). *Butyricimonas* can produce SCFA like butyric acid (Sakamoto et al., 2014) which can inhibit histone acetylase and regulate the function of intestinal macrophages to maintain intestinal homeostasis (Chang et al., 2014).

As metabolites produced by intestinal microflora fermenting dietary fiber, carbohydrates and other substances, SCFAs play a variety of physiological functions in the body on regulating metabolism, maintaining immune homeostasis and regulating inflammation (Luu and Visekruna, 2019; Blaak et al., 2020). Several studies have demonstrated that SCFAs inhibit the activity of histone deacetylase (HDAC) and play an important role in controlling inflammation, regulating the function of immune cells and promoting the repair of the colonic epithelium (Venegas et al., 2019). SCFAs promote the expression of RegIIIg and beta-defensin in intestinal epithelial cells through G-protein coupled receptor GPR43, which is essential for maintaining intestinal homeostasis (Zhao et al., 2018).

In this work, XFBD could significantly reduce the content of acetic acid (*p* < 0.05). Acetic acid is the main metabolite produced by intestinal bacteria fermenting fiber substances, which can enter the systemic circulation with the blood. Some studies reported that acetic acid can reduce inflammation and antiviral infection by activating the G-protein-coupled receptor GPR43, mediating NF-κb activation and inducing IFN-β production in lung epithelial cells (Antunes et al., 2019). However, other studies had shown that acetic acid could induce the release of pro-inflammatory cytokines, increase the calprotectin released by neutrophils under inflammatory conditions, and cause colon tissue damage due to leukocyte infiltration into the surface epithelium (Bahrami et al., 2020; Bastaki et al., 2021). Therefore, the intervention mechanism of XFBD may be related to the regulation of intestinal microecological homeostasis and the level of SCFAs, but further research is needed in the follow-up.

Additionally, the relationship between intestinal microbiota and fecal metabolites was also studied. Based on the 271 biomarkers analyzed in the MetaboAnalyst database for metabolic pathways, a total of 25 pathways were involved. These pathways mainly involved D-Glutamine and D-glutamate metabolism, Arginine Biosynthesis, Biotin metabolism, Tryptophan metabolism, Alanine, aspartate and glutamate metabolism and so on. Take the top two pathways as an example. The level of L-Glutamate and L-Citrulline increased significantly after the intervention of XFBD. Glutamate is an intermediate product of glutamine metabolism and participates in many important chemical reactions in the body, which can affect the immune response of monocytes/macrophages and the proliferation of lymphocytes (Exner et al., 2003). Glutathione (GSH) is composed of glutamate, cysteine and glycine. It can be used as a cofactor for certain antioxidant enzymes or reducing reactive oxygen species (ROS) against oxidative stress (Roth et al., 2002; Lu, 2012). Disturbance in the synthesis of glutamate and glutamine may inhibit the production of glutathione, leading to increased levels of reactive oxygen species, which in turn damages the structure and function of cell membranes, causes an inflammatory immune response, and even induces cell death (Guo et al., 2015). In Arginine Biosynthesis, Arginine is first oxidized to N-hydroxy-arginine, then convert to citrulline and release nitric oxide (NO). As an information molecule, NO plays an important role in immune and inflammatory responses (Coleman, 2001).

Finally, the results were verified by a rat model of intestinal disorder. Antibiotic treatment could change the relative abundance of microflora, destroy the intestinal barrier function and lead to the imbalance of intestinal microecological homeostasis. After the intervention of XFBD, the relative abundance of some microflora showed a pullback. It could significantly down-regulate the relative abundance of harmful bacteria such as *Bacteroides* and *Alistipes*, and up-regulate the relative abundance of beneficial bacteria such as *Ruminococcus* and *Clostridium*. The results indicate that the intervention mechanism of XFBD might be related to the regulation of intestinal flora composition. *Bacteroides* is a kind of obligate anaerobes, which can cause blood infection and abdominal abscess, and it can induce and aggravate the progress of diseases such as colitis (Rabizadeh et al., 2007). *Ruminococcus* is a gram-positive anaerobes, which plays an important role in metabolism. It can obtain nutrition by decomposing the cellulose of the host digestive system, and producing SCFAs, inhibiting the growth of harmful bacteria and protecting intestinal health (Schwiertz et al., 2002; Chassard et al., 2012).

## 5 Conclusion

In this study, UHPLC-Q-TOF/MS fecal metabolomics combined with a 16S rDNA sequencing approach was applied to evaluate the effects of XFBD on metabolomics profiling and microbial community signatures in rats. The results indicate that XFBD could improve immunity by regulating intestinal microflora. It could significantly change the relative abundance of intestinal microorganisms between phylum and genus and regulate the content of acetic acid. Also, it could partially callback the relative abundance of intestinal microflora in rats with a bacterial disorder caused by antibiotics. The evidence obtained suggests that for the treatment of COVID-19, we could carry out further research on the perspective of intestinal bacteria. Meanwhile, there are some limitations of the research. The present study is carried out based on clinical dose, multi-dose studies are needed for further research to unfold the mechanism systematically.

## Data Availability

The datasets presented in this study can be found in online repositories. The names of the repository/repositories and accession number(s) can be found in the article/Supplementary Material.
