# Supplementary material for: Modulating effect of Xuanfei Baidu granule on host metabolism and gut microbiome in rats
Source: Front Pharmacol. 2022 Sep 6;13:922642. doi: 10.3389/fphar.2022.922642 (PMC9486314; doi:10.3389/fphar.2022.922642)
Supplement: Supplementary file 1 [file DataSheet1.DOCX]

Supplementary Material

**Supplementary 1. Method S1:** Short-chain fatty acids methodological investigation.

**Supplementary 2. Figure S1:** Identification of active ingredients in XFBD. (A) The typical chromatograms of the sample at 254nm. (B) The typical chromatograms of standard compounds of 254nm. (1) hastatoside. (2) verbenalin. (3) polydatin. (4) acteoside. (5) naringin. (6) glycyrrhizic acid.

**Supplementary 3. Figure S2:** Heat map of the changing trend of the top 30 metabolites ranked by VIP value.

**Supplementary 4. Figure S3:** PCoA plot of unweighted UniFrac distance.

**Supplementary 5. Figure S4:** Venn diagram representing the overlap between control and XFBD groups.

**Supplementary 6. Figure S5:** NMDS plot of unweighted UniFrac distance.

**Supplementary 7. Figure S6:** Chao1, observe_species, Shannon and Simpson rarefaction curves in each sample.

**Supplementary 8. Figure S7:** Cladogram representation of the differentially abundant between control and XFBD.

**Supplementary 9. Table S1:** Significantly changed metabolites found in LC/MS-based metabolomic profiling.

**Supplementary 10. Table S2:** Short-chain fatty acids precision and recovery rate (n=6).

# 1 Supplementary Data

- 1. **Short-chain fatty acids Methodological Investigation**

Using mixed standard solution to investigate the instrument precision in the process of sample analysis, as shown in Table S2. The method was verified for its precision. A group of samples were randomly selected to carry out the sample recovery test according to 80%, 100% and 120% of the sample content. The experimental results were shown in the table, the results showed that the average recovery rate was between 85.91% and 109.29%, indicating that the test process could be repeated and the data obtained were stable and reliable, qualifying for metabolomics data analysis’s analytical requirements.

# 2 Supplementary Figures and Tables


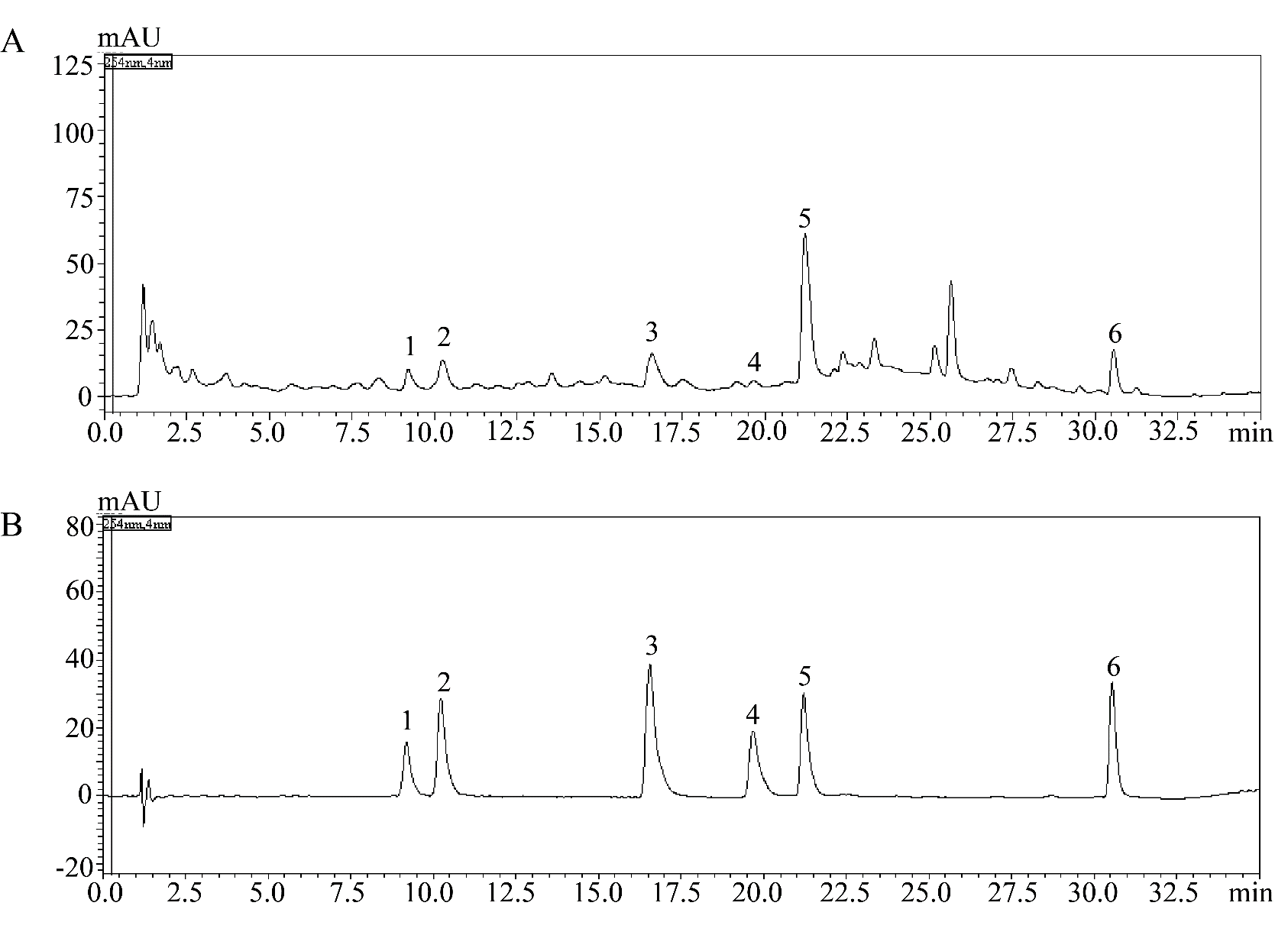


**Figure S1:** Identification of active ingredients in XFBD. (A) The typical chromatograms of the sample at 254nm. (B) The typical chromatograms of standard compounds of 254nm. (1) hastatoside. (2) verbenalin. (3) polydatin. (4) acteoside. (5) naringin. (6) glycyrrhizic acid.


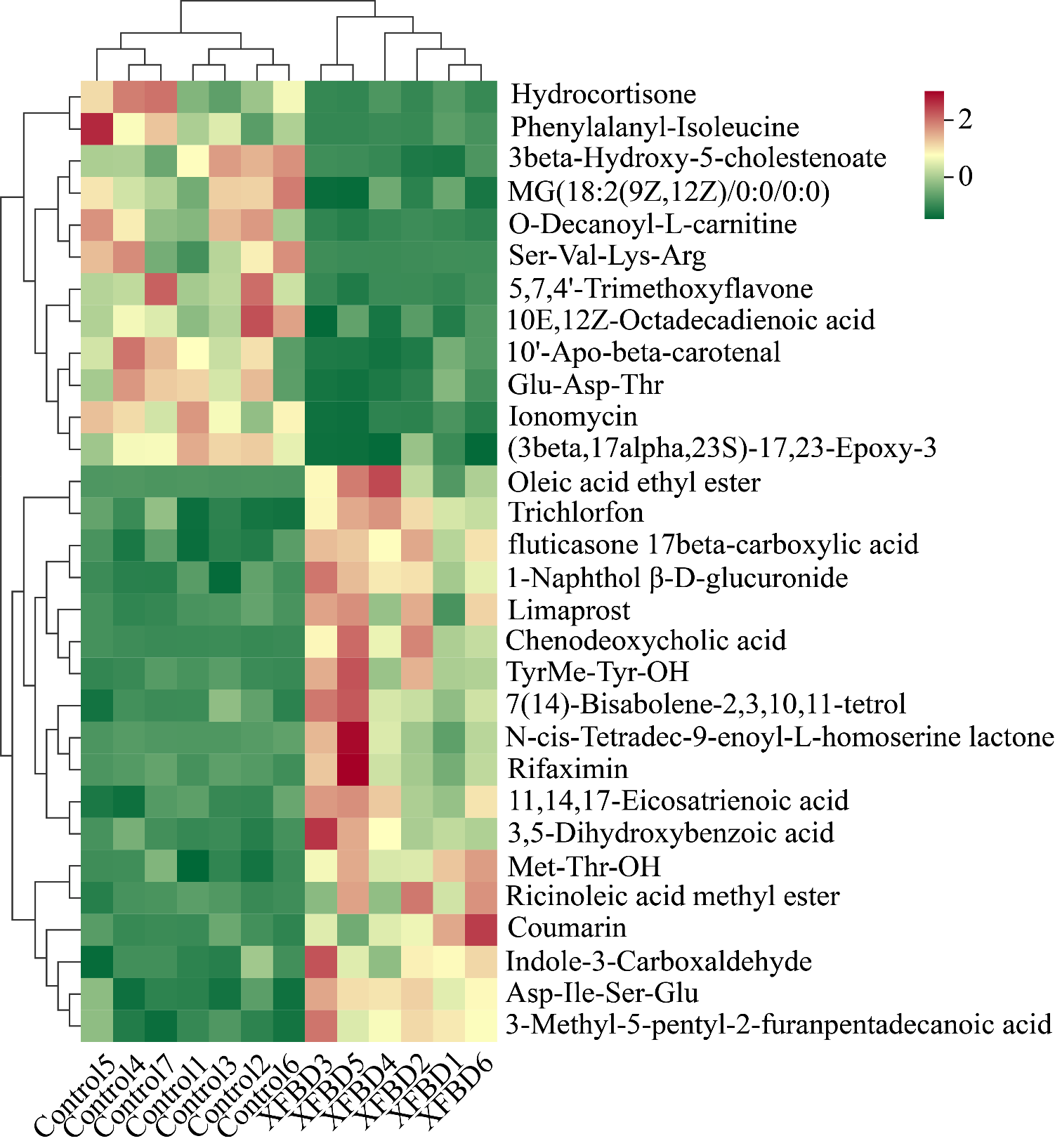


**Figure S2:** Heat map of the changing trend of the top 30 metabolites ranked by VIP value.


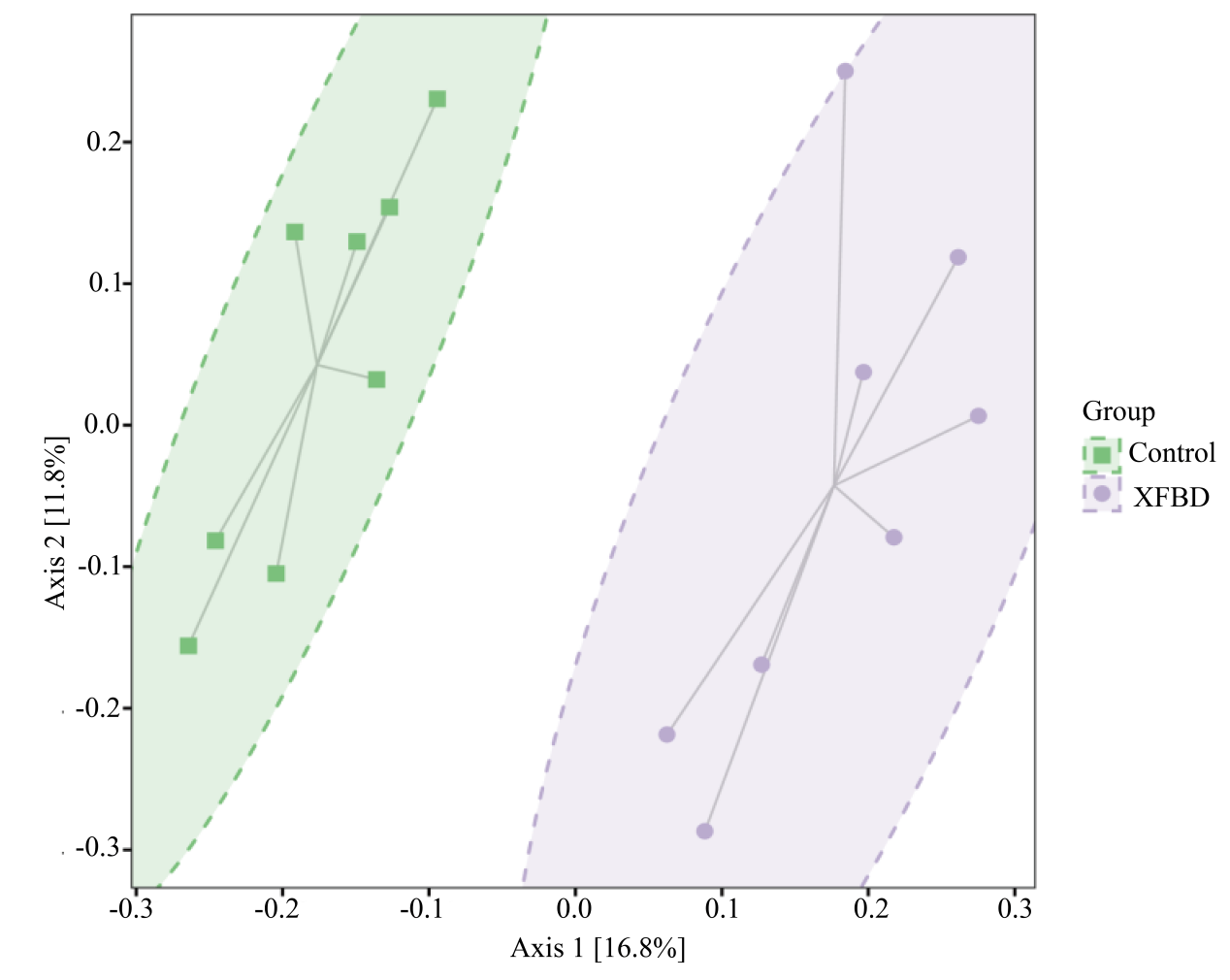


**Figure S3** PCoA plot of unweighted UniFrac distance.


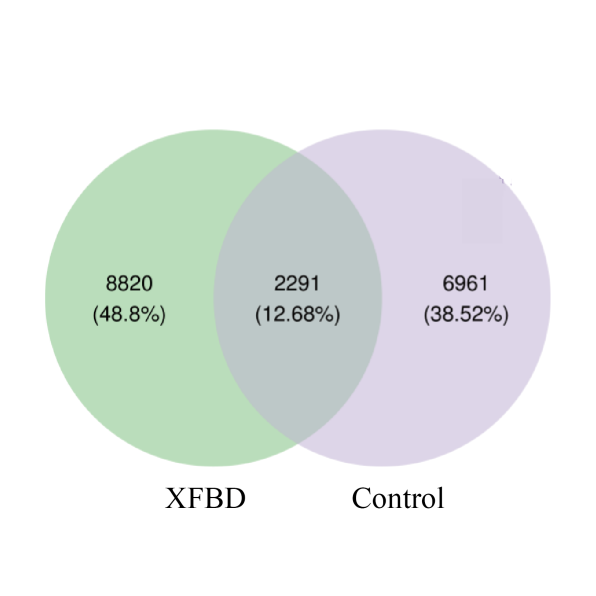


**Figure S4** Venn diagram representing the overlap between control and XFBD groups.


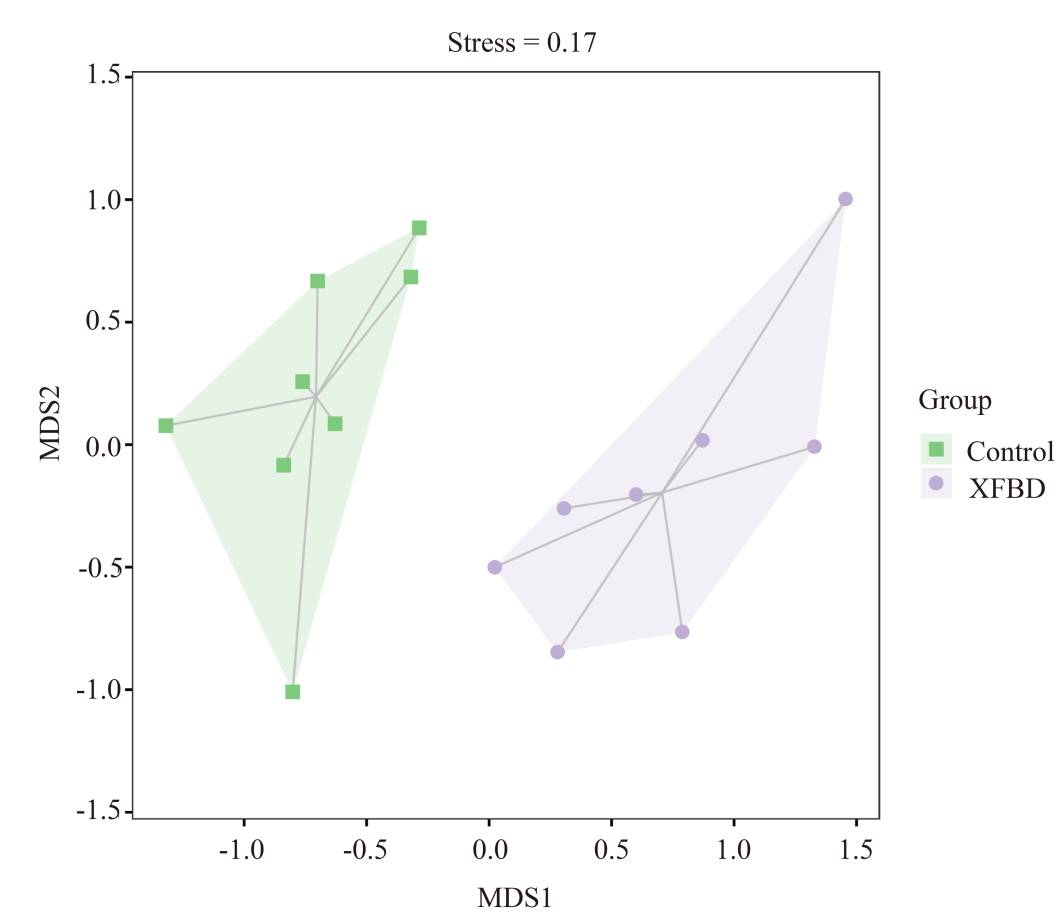


**Figure S5** NMDS plot of unweighted UniFrac distance.


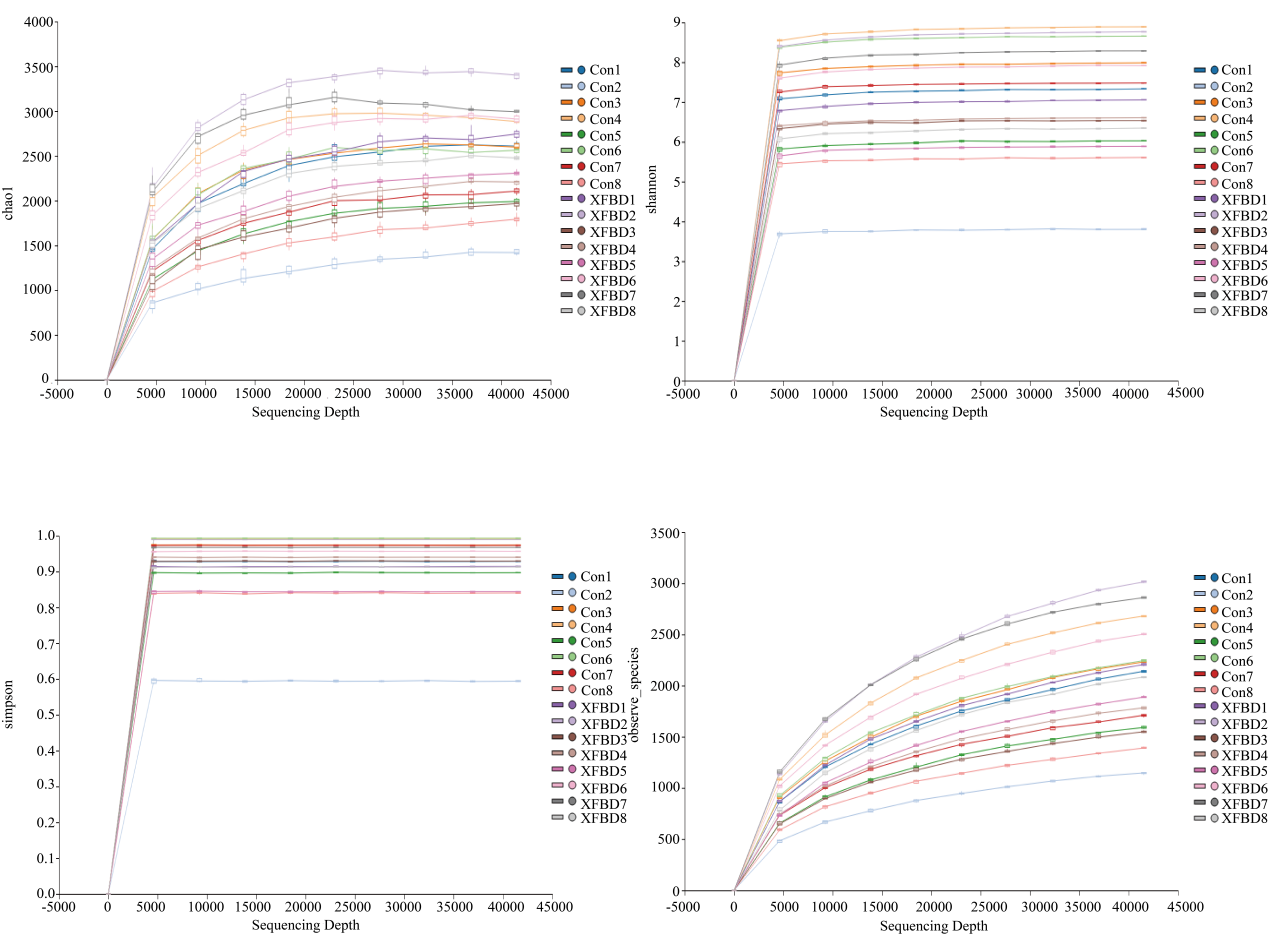


**Figure S6** Chao1, observe_species, Shannon and Simpson rarefaction curves in each sample.

**
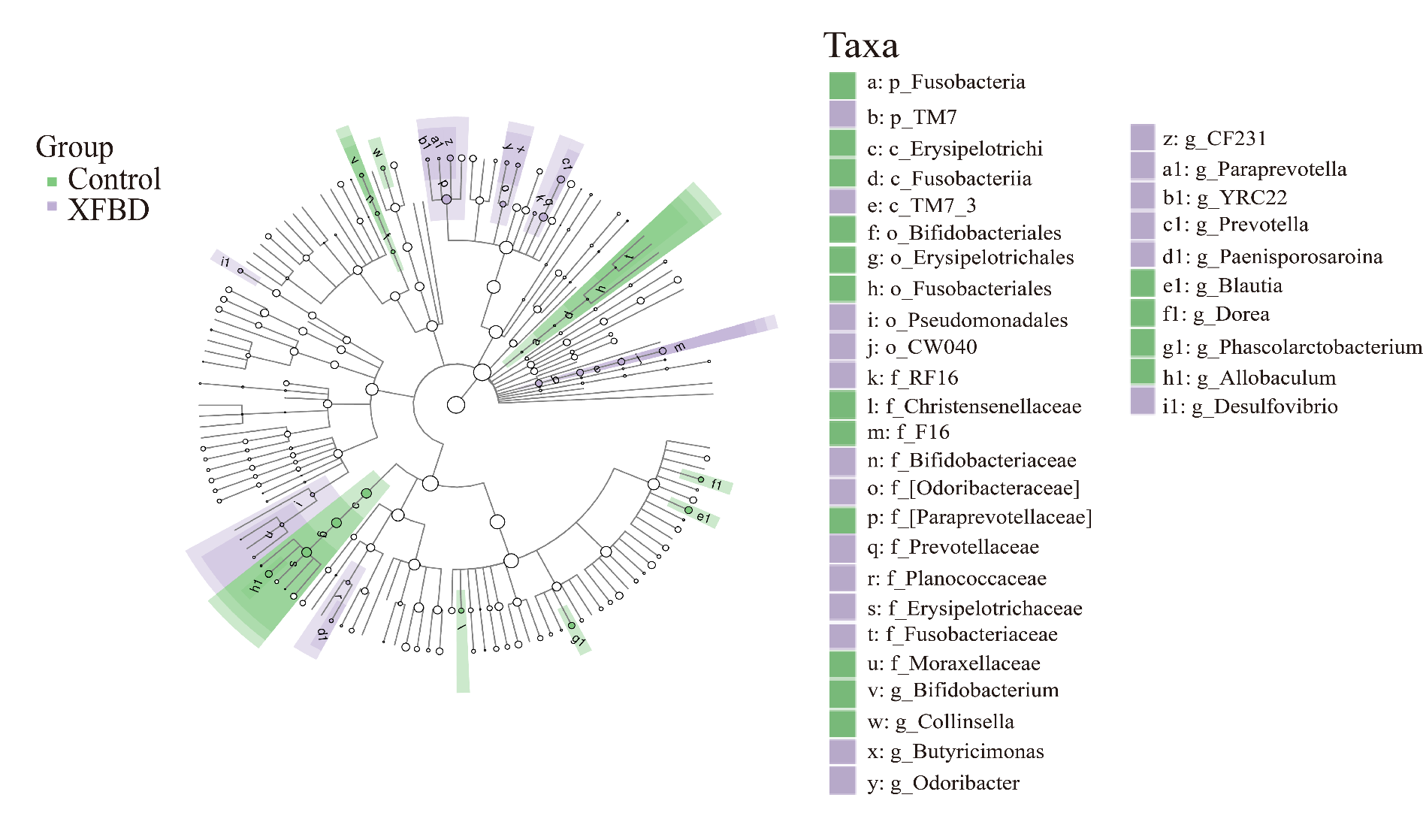
**

**Figure S7:** Cladogram representation of the differentially abundant between control and XFBD.

**Table S1** Significantly changed metabolites found in LC/MS-based metabolomic profiling.

| Metabolites | Average Rt (min) | Quant mass | Formula | VIP | P value | Log_2_(FC) | Trend |
| --- | --- | --- | --- | --- | --- | --- | --- |
| Creosol | 6.79 | 138.0681 | C8H10O2 | 1.65 | 1.62E-02 | -1.59 | down |
| 7(S),17(S)-dihydroxy-8(E),10(Z),13(Z),15(E),19(Z)-Docosapentaenoic Acid | 9.26 | 362.2457 | C22H34O4 | 1.67 | 3.41E-02 | 2.77 | up |
| 2,6-Dimethylaniline | 4.46 | 121.0891 | C8H11N | 1.34 | 2.17E-02 | 1.25 | up |
| 3'-Aenylic Acid | 1.98 | 347.0631 | C10H14N5O7P | 1.50 | 1.30E-02 | 1.61 | up |
| 5-Hydroxybisphenol A;5-Hydroxybisphenol;BPAcatechol | 8.78 | 244.1099 | C15H16O3 | 1.32 | 2.68E-02 | -1.53 | down |
| Carisoprodol | 1.62 | 260.1736 | C12H24N2O4 | 1.38 | 3.45E-02 | 1.06 | up |
| 4-Amino-2-methyl-5-pyrimidinemethanol | 1.95 | 139.0746 | C6H9N3O | 1.34 | 2.29E-03 | 1.51 | up |
| Pregnan-20-one, 17-(acetyloxy)-3-hydroxy-6-methyl-,(3a,5b,6a)- | 11.86 | 390.277 | C24H38O4 | 1.79 | 1.20E-02 | 1.12 | up |
| 5-(Diphenylphosphinyl)pentanoic acid | 5.51 | 302.1072 | C17H19O3P | 1.32 | 6.05E-03 | 1.28 | up |
| Teprenone | 15.34 | 330.2923 | C23H38O | 1.69 | 2.25E-03 | -1.67 | down |
| S-Hydroprene | 14.10 | 266.2246 | C17H30O2 | 1.61 | 2.18E-02 | -1.34 | down |
| 3beta-Hydroxy-5-cholestenoate;3beta-Hydroxycholest-5-en-27-oic acid | 10.29 | 416.329 | C27H44O3 | 2.08 | 1.19E-06 | 1.34 | up |
| 2'-Deoxyadenosine-5'-Monophosphate | 2.15 | 331.0682 | C10H14N5O6P | 1.25 | 1.21E-02 | 1.48 | up |
| 4-Methylbenzenemethanol | 4.46 | 122.0732 | C8H10O | 1.37 | 1.01E-02 | 1.91 | up |
| Glu-Val | 3.31 | 246.1216 | C10H18N2O5 | 1.29 | 4.37E-02 | 2.06 | up |
| 4-Methoxyestrone | 8.42 | 300.1725 | C19H24O3 | 1.66 | 1.91E-02 | -3.67 | down |
| 1-Hydroxy-2-naphthaldehyde | 8.90 | 172.0524 | C11H8O2 | 1.61 | 7.93E-03 | -1.25 | down |
| D-(+)-sucrose | 1.94 | 342.1162 | C12H22O11 | 1.55 | 1.81E-02 | -1.12 | down |
| 5α-Pregnane-3α,21-diol-20-one | 16.28 | 334.2508 | C21H34O3 | 1.45 | 1.58E-02 | -2.06 | down |
| 5,7,4'-Trimethoxyflavone | 7.06 | 312.0998 | C18H16O5 | 2.06 | 3.01E-03 | -1.64 | down |
| Ionomycin | 18.07 | 708.5176 | C41H72O9 | 2.00 | 1.80E-03 | -1.98 | down |
| 3-Hydroxyanthranilic acid | 6.70 | 153.0426 | C7H7NO3 | 1.24 | 3.88E-02 | -1.75 | down |
| γ-Muricholic acid | 12.64 | 408.2876 | C24H40O5 | 1.82 | 1.84E-02 | 1.58 | up |
| Doxercalciferol | 16.95 | 412.3341 | C28H44O2 | 1.48 | 3.91E-02 | 1.40 | up |
| Taurocyamine | 6.31 | 167.0365 | C3H9N3O3S | 1.33 | 1.70E-02 | -1.18 | down |
| 2-Arachidonoylglycerol | 14.36 | 378.277 | C23H38O4 | 1.62 | 1.81E-02 | 1.04 | up |
| Alitretinoin | 13.11 | 300.2089 | C20H28O2 | 1.38 | 6.77E-03 | 1.53 | up |
| Trichlorfon | 7.21 | 255.9226 | C4H8Cl3O4P | 1.97 | 8.37E-05 | 1.64 | up |
| Aldosterone | 5.70 | 360.1937 | C21H28O5 | 1.80 | 5.36E-03 | 2.13 | up |
| Etofylline | 3.86 | 224.0909 | C9H12N4O3 | 1.36 | 8.26E-03 | 1.86 | up |
| 12-Ketodeoxycholic acid | 12.63 | 390.277 | C24H38O4 | 1.82 | 2.79E-02 | 1.45 | up |
| Soyasapogenol A | 12.19 | 474.3709 | C30H50O4 | 1.53 | 3.50E-02 | -1.42 | down |
| N-Phenylacetylglycine | 6.77 | 193.0739 | C10H11NO3 | 1.47 | 1.83E-02 | -2.04 | down |
| D-Sorbitol | 6.62 | 182.079 | C6H14O6 | 1.65 | 4.69E-02 | 2.80 | up |
| Hydrocortisone | 12.90 | 362.2093 | C21H30O5 | 2.19 | 2.64E-03 | 4.54 | up |
| Indole-3-Carboxaldehyde | 4.90 | 145.0528 | C9H7NO | 1.88 | 9.98E-04 | -2.27 | down |
| Chaksine | 8.43 | 450.2955 | C22H38N6O4 | 1.72 | 1.90E-03 | 1.85 | up |
| Chenodeoxycholic acid | 14.51 | 392.2927 | C24H40O4 | 1.94 | 4.02E-02 | 3.28 | up |
| 2-Propenamide, 3-(4-chlorophenyl)-N-(3-methoxyphenyl)- | 4.96 | 287.0713 | C16H14ClNO2 | 1.34 | 2.28E-02 | -1.81 | down |
| 7alpha,27-Dihydroxycholesterol | 12.54 | 418.3447 | C27H46O3 | 1.54 | 2.67E-02 | 1.23 | up |
| Betulinic acid | 13.33 | 456.3603 | C30H48O3 | 1.39 | 3.87E-02 | -1.23 | down |
| (+)-trans-Chrysanthemic acid | 6.30 | 168.115 | C10H16O2 | 1.63 | 8.45E-03 | 1.06 | up |
| Betulonic acid | 13.01 | 454.3447 | C30H46O3 | 1.65 | 7.40E-03 | -1.52 | down |
| Harmine | 2.19 | 212.095 | C13H12N2O | 1.40 | 4.62E-03 | 1.56 | up |
| Methoprene;(E,E)-1-Methylethyl 11-methoxy-3,7,11-trimethyl-2,4-dodecadienoate | 15.84 | 310.2508 | C19H34O3 | 1.31 | 3.54E-02 | -1.35 | down |
| Adrenosterone | 4.71 | 300.1725 | C19H24O3 | 1.75 | 2.87E-03 | 1.55 | up |
| Ifosfamide | 3.48 | 260.0248 | C7H15Cl2N2O2P | 1.67 | 1.91E-02 | -1.24 | down |
| L-Citrulline | 1.96 | 175.0957 | C6H13N3O3 | 1.27 | 5.04E-03 | 1.49 | up |
| 2',4'-Dihydroxy-6'-methoxyacetophenone | 4.99 | 182.0579 | C9H10O4 | 1.79 | 1.21E-02 | -1.74 | down |
| DL-Pipecolic Acid | 1.73 | 129.079 | C6H11NO2 | 1.44 | 2.36E-03 | 1.34 | up |
| Ursocholic acid | 11.54 | 408.2876 | C24H40O5 | 1.59 | 1.14E-02 | 1.75 | up |
| Dodecylbenzenesulfonic acid | 5.69 | 326.1916 | C18H30O3S | 1.61 | 1.37E-02 | 2.85 | up |
| 9,10-DiHOME | 10.19 | 314.2457 | C18H34O4 | 1.46 | 1.59E-02 | -1.24 | down |
| N-Oleoyl Glycine | 14.50 | 339.2773 | C20H37NO3 | 1.78 | 4.17E-02 | 2.48 | up |
| Glu-Leu | 4.86 | 260.1372 | C11H20N2O5 | 1.81 | 5.03E-04 | 2.07 | up |
| Mycophenolic acid | 7.23 | 320.126 | C17H20O6 | 1.73 | 5.96E-03 | 1.94 | up |
| 3-Hydroxylanosta-8,24-dien-21-oic acid | 13.33 | 456.3603 | C30H48O3 | 1.45 | 3.22E-02 | -1.28 | down |
| Crepenynate;(9Z)-Octadec-9-en-12-ynoate;(Z)-9-Octadecen-12-ynoic acid;Crepenynic acid | 10.19 | 278.2246 | C18H30O2 | 1.61 | 2.79E-03 | -1.33 | down |
| Bisabolol oxide A | 16.77 | 238.1933 | C15H26O2 | 1.38 | 4.46E-02 | -1.41 | down |
| N-Desmethylclobazam | 2.16 | 286.0509 | C15H11ClN2O2 | 1.47 | 3.45E-02 | 1.36 | up |
| 7-Methoxyflavone | 8.78 | 252.0786 | C16H12O3 | 1.35 | 1.96E-02 | -1.61 | down |
| N-Formiminoglycine;Formiminoglycine;N-Formimidoylglycine | 6.86 | 102.0429 | C3H6N2O2 | 1.40 | 3.83E-02 | -1.09 | down |
| 25-Hydroxycholesterol | 16.38 | 402.3498 | C27H46O2 | 1.76 | 3.82E-02 | 1.02 | up |
| 6-O-methylguanine | 7.07 | 165.0651 | C6H7N5O | 1.54 | 6.56E-03 | -1.82 | down |
| Quinoline-2-carboxylic acid | 6.29 | 173.0477 | C10H7NO2 | 1.34 | 9.55E-03 | -1.24 | down |
| Betalamic acid | 8.79 | 211.0481 | C9H9NO5 | 1.30 | 3.06E-02 | -1.51 | down |
| 2-Methylamino-1-(3,4-methylenedioxyphenyl)propan-1-one | 5.12 | 207.0895 | C11H13NO3 | 1.34 | 1.81E-02 | 1.08 | up |
| 5β-Androstane-3α,17β-diol | 18.13 | 292.2402 | C19H32O2 | 1.66 | 1.20E-02 | -1.50 | down |
| 3-Hydroxymandelic acid | 2.02 | 168.0423 | C8H8O4 | 1.14 | 1.68E-02 | 1.14 | up |
| Aprepitant | 2.17 | 534.1502 | C23H21F7N4O3 | 1.32 | 3.13E-02 | 1.71 | up |
| 11,14,17-Eicosatrienoic acid | 15.01 | 306.2559 | C20H34O2 | 1.91 | 1.99E-03 | -1.09 | down |
| 3-Ethylmethcathinone | 15.61 | 191.131 | C12H17NO | 1.73 | 4.06E-02 | -2.30 | down |
| 7,10,13,16-Docosatetraenoylethanolamine | 13.60 | 375.3137 | C24H41NO2 | 1.43 | 7.13E-03 | 1.06 | up |
| Dihydrocortisol | 5.70 | 364.225 | C21H32O5 | 1.70 | 2.70E-02 | 1.46 | up |
| 2-Benzyl-4-(octahydro-2H-isoindol-2-yl)-4-oxobutanoic acid | 8.37 | 315.1834 | C19H25NO3 | 1.63 | 1.39E-02 | 1.20 | up |
| 1-(1-Methylindol-5-yl)-3-(3-pyridyl)urea | 6.31 | 266.1168 | C15H14N4O | 1.60 | 8.44E-03 | 1.31 | up |
| Ricinoleic acid methyl ester | 16.34 | 312.2664 | C19H36O3 | 1.89 | 3.75E-05 | -2.65 | down |
| Diisooctyl phthalate | 12.61 | 390.277 | C24H38O4 | 1.77 | 3.46E-02 | 2.05 | up |
| 17-Epiestriol | 5.18 | 288.1725 | C18H24O3 | 1.78 | 3.71E-03 | 1.76 | up |
| Indoleacrylic acid | 4.90 | 187.0633 | C11H9NO2 | 1.73 | 2.58E-03 | -2.19 | down |
| 4',5,7-Trimethoxyisoflavone | 8.78 | 312.0998 | C18H16O5 | 1.30 | 2.06E-02 | -1.62 | down |
| 5-Norbornene-2,3-dicarboximide, N-(2-ethylhexyl)- | 6.41 | 275.1885 | C17H25NO2 | 1.46 | 3.73E-02 | 1.50 | up |
| Oleic acid ethyl ester | 16.59 | 310.2872 | C20H38O2 | 1.98 | 8.55E-03 | 1.43 | up |
| Piperidine | 8.90 | 85.0891 | C5H11N | 1.67 | 3.64E-03 | -1.09 | down |
| 13(S)-HODE methyl ester | 15.59 | 310.2508 | C19H34O3 | 1.43 | 2.17E-02 | 2.02 | up |
| Tacrolimus | 12.62 | 803.482 | C44H69NO12 | 1.77 | 7.59E-03 | 1.53 | up |
| Atipamezole | 10.18 | 212.1313 | C14H16N2 | 1.55 | 2.31E-02 | 1.72 | up |
| Coumarin | 4.90 | 146.0368 | C9H6O2 | 1.89 | 1.04E-03 | -2.40 | down |
| N-Acetyl-D-phenylalanine | 8.90 | 207.0895 | C11H13NO3 | 1.54 | 7.04E-03 | -1.31 | down |
| Cycloxydime | 5.70 | 325.1712 | C17H27NO3S | 1.59 | 8.28E-03 | 2.13 | up |
| 9(R)-HODE | 11.26 | 296.2351 | C18H32O3 | 1.26 | 3.49E-02 | 1.29 | up |
| N-Methylaniline | 8.91 | 107.0735 | C7H9N | 1.70 | 4.24E-03 | -1.62 | down |
| cis-4,10-13,16-Docosatetraenoic Acid methyl ester | 14.86 | 346.2872 | C23H38O2 | 1.59 | 1.24E-02 | 1.88 | up |
| beta-D-4-Deoxy-Delta4-GlcA-(1->4)-beta-D-Glc-(1->4)-alpha-L-Rha-(1->3)-D-Glc;beta-D-4-Deoxy-Delta4-GlcAp-(1->4)-beta-D-Glcp-(1->4)-alpha-L-Rhap-(1->3)-D-Glcp | 2.14 | 646.1956 | C24H38O20 | 1.24 | 2.58E-02 | -1.38 | down |
| [(1R,4Br,5R,10aS,12aR)-1-(furan-3-yl)-4b,7,7,10a,12a-pentamethyl-3-oxo-1,5,6,6a,8,9,10,10b,11,12-decahydronaphtho[2,1-f]isochromen-5-yl] acetate | 8.02 | 454.2719 | C28H38O5 | 1.49 | 3.71E-02 | 2.81 | up |
| Val-Leu | 4.94 | 230.163 | C11H22N2O3 | 1.54 | 2.10E-02 | -1.21 | down |
| Val-Glu-Tyr-His-Gln | 10.40 | 674.3024 | C30H42N8O10 | 1.69 | 4.47E-02 | 2.29 | up |
| Val-Arg-Gln-Val-Asp | 15.09 | 615.334 | C25H45N9O9 | 1.79 | 3.10E-03 | 1.24 | up |
| Tyr-Gln-Ile-Arg | 15.65 | 578.3176 | C26H42N8O7 | 1.54 | 2.84E-03 | 1.32 | up |
| Thr-Leu-Asn | 10.02 | 346.1852 | C14H26N4O6 | 1.64 | 4.24E-02 | 1.35 | up |
| Spinorphin, bovine | 12.60 | 876.4745 | C45H64N8O10 | 1.56 | 2.73E-02 | -2.17 | down |
| Ser-Val-Lys-Arg | 10.11 | 488.3071 | C20H40N8O6 | 2.06 | 3.61E-05 | 1.45 | up |
| Ser-His-Val-Lys | 8.38 | 469.2649 | C20H35N7O6 | 1.68 | 1.16E-02 | 1.09 | up |
| Pro-Ser-Ser | 2.10 | 289.1274 | C11H19N3O6 | 1.66 | 8.43E-03 | 1.16 | up |
| Pro-Lys-Lys | 9.42 | 371.2533 | C17H33N5O4 | 1.84 | 7.60E-03 | 1.13 | up |
| Phenylalanyl-Isoleucine | 5.14 | 278.163 | C15H22N2O3 | 1.81 | 1.14E-02 | 2.68 | up |
| Phe-Tyr-Ser | 2.71 | 415.1743 | C21H25N3O6 | 1.36 | 3.15E-02 | 2.00 | up |
| Perindopril | 12.95 | 368.2311 | C19H32N2O5 | 1.55 | 1.00E-02 | 1.22 | up |
| PACLITAXEL | 9.68 | 853.331 | C47H51NO14 | 1.52 | 1.19E-02 | -1.27 | down |
| Nap-TyrMe-OH | 3.68 | 500.1584 | C28H24N2O7 | 1.48 | 4.98E-03 | 2.40 | up |
| N-cis-Tetradec-9-enoyl-L-homoserine lactone | 11.89 | 309.2304 | C18H31NO3 | 1.92 | 3.45E-02 | 3.06 | up |
| N-benzyl-1-methyl-1H-pyrazolo[3,4-d]pyrimidin-4-amine | 4.85 | 239.1171 | C13H13N5 | 1.48 | 3.47E-02 | 1.12 | up |
| N-Demethylnarwedine | 2.17 | 271.1208 | C16H17NO3 | 1.50 | 3.24E-02 | 1.02 | up |
| N-Acetylindoxyl;Acetylindoxyl | 7.94 | 175.0633 | C10H9NO2 | 1.67 | 2.44E-03 | -2.28 | down |
| Met-Lys-Glu | 7.98 | 406.1886 | C16H30N4O6S1 | 1.82 | 4.14E-02 | -3.04 | down |
| MG(18:2(9Z,12Z)/0:0/0:0) | 13.70 | 354.277 | C21H38O4 | 2.07 | 4.32E-05 | 1.34 | up |
| Lys-Lys-Val | 12.63 | 373.2689 | C17H35N5O4 | 1.64 | 2.59E-02 | 1.61 | up |
| Lys-Leu-His-Val-Asp | 7.96 | 610.3439 | C27H46N8O8 | 1.48 | 7.97E-03 | 1.06 | up |
| L-N2-(2-Carboxyethyl)arginine;N2-(2-Carboxyethyl)-L-arginine | 8.31 | 246.1328 | C9H18N4O4 | 1.37 | 2.72E-02 | -1.06 | down |
| Ile-Phe-Gln-Glu | 6.11 | 535.2642 | C25H37N5O8 | 1.33 | 2.72E-02 | -1.11 | down |
| Homodolicholide | 18.43 | 492.3451 | C29H48O6 | 1.56 | 1.28E-02 | -3.70 | down |
| His-Gln-Val-Lys | 5.14 | 510.2914 | C22H38N8O6 | 1.03 | 1.44E-02 | -1.92 | down |
| Glu-Asp-Thr | 6.29 | 363.1278 | C13H21N3O9 | 2.00 | 2.29E-02 | 5.46 | up |
| FFA(22:7) | 8.91 | 326.2246 | C22H30O2 | 1.72 | 3.05E-02 | 1.70 | up |
| Ethyl 3-hydroxyoctanoate | 6.40 | 188.1412 | C10H20O3 | 1.43 | 1.88E-02 | -1.08 | down |
| Ethenodeoxyadenosine | 2.69 | 275.1018 | C12H13N5O3 | 1.24 | 9.72E-03 | 1.77 | up |
| DG(18:1n7/0:0/18:4n3) | 17.36 | 614.491 | C39H66O5 | 1.22 | 4.66E-02 | -2.91 | down |
| DG(16:1n7/0:0/22:6n3) | 14.63 | 638.491 | C41H66O5 | 1.33 | 3.49E-02 | 1.16 | up |
| Caldariellaquinol | 18.23 | 632.4661 | C39H68O2S2 | 1.44 | 4.35E-02 | -1.17 | down |
| Bismurrayafoline E | 15.37 | 724.424 | C48H56N2O4 | 1.44 | 2.47E-02 | 1.72 | up |
| Asp-Val-Glu | 3.91 | 361.1485 | C14H23N3O8 | 1.71 | 5.89E-03 | 1.50 | up |
| Arg-Ile-Ile | 15.61 | 400.2798 | C18H36N6O4 | 1.73 | 2.35E-02 | -2.52 | down |
| Arg-His-His | 6.29 | 448.2295 | C18H28N10O4 | 1.36 | 3.59E-02 | -4.13 | down |
| Antibiotic SB 202742 | 10.38 | 370.2508 | C24H34O3 | 1.62 | 6.11E-03 | 1.61 | up |
| 7alpha-hydroxy-3-oxochol-4-en-24-oic Acid | 11.53 | 388.2614 | C24H36O4 | 1.68 | 1.24E-02 | 1.42 | up |
| 5,7-dihydroxy-6,8-bis[3,4,5-trihydroxy-6-(hydroxymethyl)oxan-2-yl]-2-(2,4,5-trihydroxyphenyl)-4H-chromen-4-one | 2.19 | 626.1483 | C27H30O17 | 1.50 | 4.79E-02 | -1.26 | down |
| 5,6 epoxy-cholesterol | 16.79 | 402.3498 | C27H46O2 | 1.46 | 3.32E-02 | 1.12 | up |
| 3-Hydroxylanosta-7,24-dien-21-oic acid | 14.19 | 456.3603 | C30H48O3 | 1.51 | 4.41E-02 | 1.53 | up |
| 3,7,12-Trihydroxycholan-24-oic acid (stereoisomer unknown) | 12.16 | 408.2876 | C24H40O5 | 1.13 | 4.38E-03 | 1.06 | up |
| 20a,22b-Dihydroxycholesterol | 14.01 | 418.3447 | C27H46O3 | 1.59 | 9.40E-03 | 1.20 | up |
| 2-Amino-2-deoxyisochorismate;(2S)-2-Amino-4-deoxychorismate | 7.76 | 225.0637 | C10H11NO5 | 1.39 | 1.64E-02 | 1.02 | up |
| 2-(2,6-DIMETHOXYPHENOXYETHYL)AMINOMETHYL-1,4-BENZODIOXANE(WB 4101) | 4.68 | 345.1576 | C19H23NO5 | 1.72 | 7.80E-04 | 1.18 | up |
| 1H-Indole-3,5-dicarboxylic acid, 1-[3-[4-(decyloxy)phenoxy]-2-oxopropyl]-, 3-methyl ester | 6.39 | 523.257 | C30H37NO7 | 1.14 | 2.69E-02 | -2.70 | down |
| 19(20)-Epoxy-4Z,7Z,10Z,13Z,16Z-docosapentaenoic acid | 8.91 | 344.2351 | C22H32O3 | 1.71 | 3.31E-02 | 1.71 | up |
| 15-keto Latanoprost | 11.85 | 430.2719 | C26H38O5 | 1.80 | 4.94E-02 | 2.31 | up |
| 15,15'-Dihydroxy-beta-carotene | 15.30 | 570.4437 | C40H58O2 | 1.63 | 2.21E-02 | 1.08 | up |
| 13'-Hydroxy-alpha-tocotrienol | 14.00 | 440.329 | C29H44O3 | 1.42 | 4.99E-02 | 1.20 | up |
| 12(13)-EpOME;(12R,13S)-(9Z)-12,13-Epoxyoctadecenoic acid | 10.19 | 296.2351 | C18H32O3 | 1.73 | 1.10E-03 | -1.64 | down |
| 1-Phenyl-1,3-octadecanedione | 14.33 | 358.2872 | C24H38O2 | 1.42 | 4.37E-02 | 1.02 | up |
| (8E,10R,12Z)-10-Hydroperoxy-8,12-octadecadienoate | 10.68 | 312.2301 | C18H32O4 | 1.84 | 4.16E-04 | -2.29 | down |
| (3beta,5alpha,9alpha,22E,24R)-3,5,9-Trihydroxy-23-methylergosta-7,22-dien-6-one | 14.01 | 458.3396 | C29H46O4 | 1.78 | 4.23E-03 | 1.82 | up |
| (24S)-24,25-dihydroxyvitamin D3 | 16.63 | 416.329 | C27H44O3 | 1.72 | 1.48E-02 | -1.95 | down |
| (23R)-23,25-dihydroxyvitamin D3 | 12.05 | 416.329 | C27H44O3 | 1.56 | 1.83E-03 | 1.18 | up |
| (1E,4S,5E,7R)-Germacra-1(10),5-dien-11-ol;Germacradienol | 5.84 | 222.1984 | C15H26O | 1.74 | 7.72E-03 | -1.05 | down |
| 1H-Benzimidazole-7-carboxamide, 2-[(2R)-2-methyl-2-pyrrolidinyl]- | 6.30 | 244.1324 | C13H16N4O | 1.59 | 8.81E-03 | 1.27 | up |
| Melilotocarpan C | 8.77 | 330.1103 | C18H18O6 | 1.20 | 2.18E-02 | -1.55 | down |
| cholic acid | 9.57 | 362.2093 | C21H30O5 | 1.24 | 2.43E-02 | 1.25 | up |
| 3',4'-Dimethoxy-2'-hydroxychalcone | 7.39 | 284.1049 | C17H16O4 | 1.36 | 3.42E-02 | 1.37 | up |
| Limaprost | 9.25 | 380.2563 | C22H36O5 | 1.96 | 7.59E-03 | 2.23 | up |
| 4,4'-Dihydroxy-alpha-methylstilbene | 9.30 | 226.0994 | C15H14O2 | 1.75 | 2.65E-02 | 1.26 | up |
| Desmethyldeschlorobenzoyl Indomethacin | 6.29 | 191.0582 | C10H9NO3 | 1.44 | 7.26E-03 | -1.35 | down |
| L-DOPA methyl ester | 6.82 | 211.0845 | C10H13NO4 | 1.62 | 7.31E-03 | -2.19 | down |
| (R-(R*,R*))-3-(3-Cyclohexyl-3-hydroxypropyl)-2,5-dioxo-imidazolidine-4-heptanoic acid | 12.96 | 368.2311 | C19H32N2O5 | 1.24 | 1.03E-02 | 1.32 | up |
| Glycoursodeoxycholic acid | 11.28 | 449.3141 | C26H43NO5 | 1.49 | 1.34E-02 | 1.78 | up |
| N-Stearoyltaurine | 12.14 | 391.2756 | C20H41NO4S | 1.27 | 3.91E-02 | 1.63 | up |
| 2-Hydroxycinnamic acid | 7.39 | 164.0473 | C9H8O3 | 1.33 | 3.01E-02 | 1.02 | up |
| Staphidine | 15.49 | 606.4549 | C42H58N2O | 1.50 | 1.47E-02 | -1.17 | down |
| 1-Palmitoyl-2-docosahexaenoyl-sn-glycero-3-phosphocholine | 11.48 | 805.5622 | C46H80NO8P | 1.56 | 2.95E-02 | 2.15 | up |
| Matairesinol | 9.80 | 358.1416 | C20H22O6 | 1.65 | 1.47E-02 | -3.28 | down |
| Biocytin | 9.19 | 372.1831 | C16H28N4O4S | 1.62 | 2.93E-04 | 1.72 | up |
| L-Glutamic Acid | 1.88 | 147.0532 | C5H9NO4 | 1.67 | 1.01E-02 | 1.85 | up |
| 11-deoxy-16,16-dimethyl-PGE2 | 10.20 | 364.2614 | C22H36O4 | 1.58 | 4.96E-02 | 1.86 | up |
| 8-iso-16-cyclohexyl-tetranor Prostaglandin E2 | 9.63 | 378.2406 | C22H34O5 | 1.67 | 4.71E-02 | 2.46 | up |
| Digitogenin | 14.74 | 448.3189 | C27H44O5 | 1.31 | 3.47E-02 | 1.26 | up |
| 1,6-Di-O-phosphono-D-fructose | 5.80 | 339.9961 | C6H14O12P2 | 1.35 | 4.24E-02 | 1.74 | up |
| Artemisin | 9.34 | 262.1205 | C15H18O4 | 1.46 | 4.32E-03 | 1.26 | up |
| Naringenin | 7.62 | 272.0685 | C15H12O5 | 1.57 | 2.18E-02 | -1.22 | down |
| Ubiquinone-4 | 14.41 | 454.3083 | C29H42O4 | 1.57 | 1.50E-02 | 1.10 | up |
| 1-O-Cinnamoylglucose | 7.07 | 310.1053 | C15H18O7 | 1.35 | 6.20E-03 | -1.23 | down |
| O-Decanoyl-L-carnitine | 11.61 | 315.241 | C17H33NO4 | 2.06 | 1.92E-03 | -3.62 | down |
| Penciclovir | 15.37 | 253.1175 | C10H15N5O3 | 1.44 | 2.36E-02 | 1.45 | up |
| Azatadine | 7.27 | 290.1783 | C20H22N2 | 1.61 | 1.15E-02 | 1.21 | up |
| 7-Hydroxyamoxapine | 7.40 | 329.0931 | C17H16ClN3O2 | 1.27 | 3.16E-02 | 1.07 | up |
| Glycodeoxycholic acid | 14.74 | 449.3141 | C26H43NO5 | 1.33 | 3.80E-02 | 1.19 | up |
| 2',6'-Dihydroxy 4,4'-dimethoxydihydrochalcone | 7.25 | 302.1154 | C17H18O5 | 1.34 | 4.74E-03 | 1.49 | up |
| Stercobilin | 8.36 | 594.3417 | C33H46N4O6 | 1.64 | 1.35E-02 | 1.26 | up |
| O-Desmethylmycophenolic acid | 5.63 | 306.1103 | C16H18O6 | 1.83 | 1.11E-02 | 1.23 | up |
| Allopurinol | 3.10 | 136.0385 | C5H4N4O | 1.38 | 3.72E-02 | 1.76 | up |
| fluticasone 17beta-carboxylic acid | 4.90 | 396.1748 | C21H26F2O5 | 1.97 | 4.83E-04 | 1.75 | up |
| 15-Keto-prostaglandin E2 | 8.86 | 350.2093 | C20H30O5 | 1.72 | 1.52E-02 | 2.41 | up |
| Propanoic acid, 2-[[4-[2-[[[(2,4-difluorophenyl)amino]carbonyl]heptylamino]ethyl]phenyl]thio]-2-methyl- | 11.25 | 492.2258 | C26H34F2N2O3S | 1.23 | 4.31E-02 | 1.59 | up |
| 3α,7α-Dihydroxy-12-oxocholanoic acid | 11.36 | 406.2719 | C24H38O5 | 1.63 | 8.48E-03 | 1.48 | up |
| 10E,12Z-Octadecadienoic acid | 14.10 | 280.2402 | C18H32O2 | 2.05 | 7.09E-05 | -2.35 | down |
| Batrachotoxin | 11.52 | 538.3043 | C31H42N2O6 | 1.69 | 5.04E-03 | 2.27 | up |
| Aspartame | 5.28 | 294.1216 | C14H18N2O5 | 1.69 | 2.79E-04 | 1.68 | up |
| Allocholic acid | 11.53 | 376.2977 | C24H40O3 | 1.52 | 1.90E-02 | 2.01 | up |
| Eicosapentaenoyl Serotonin | 15.21 | 460.309 | C30H40N2O2 | 1.79 | 3.33E-04 | -1.15 | down |
| 3,5-Dihydroxybenzoic acid | 5.60 | 154.0266 | C7H6O4 | 1.91 | 8.24E-03 | -3.85 | down |
| 5-Acetylamino-6-amino-3-methyluracil | 1.87 | 198.0753 | C7H10N4O3 | 1.79 | 3.21E-03 | 1.46 | up |
| Aatrex | 4.94 | 215.0938 | C8H14ClN5 | 1.76 | 2.96E-02 | 1.95 | up |
| Callystatin A | 14.97 | 456.324 | C29H44O4 | 1.74 | 6.40E-04 | 1.73 | up |
| N-Arachidonoyl-L-Serine | 12.63 | 391.2723 | C23H37NO4 | 1.82 | 2.31E-02 | 1.42 | up |
| 1-Naphthol β-D-glucuronide | 5.46 | 320.0896 | C16H16O7 | 1.96 | 1.61E-03 | 1.10 | up |
| Tryptophan | 4.92 | 204.0899 | C11H12N2O2 | 1.61 | 3.46E-03 | -2.42 | down |
| Albizziin | 6.29 | 147.0644 | C4H9N3O3 | 1.44 | 8.35E-03 | -1.11 | down |
| 4-Guanidinobutyric acid | 5.44 | 145.0851 | C5H11N3O2 | 1.74 | 1.55E-03 | 1.23 | up |
| Nevirapine | 6.31 | 266.1168 | C15H14N4O | 1.73 | 1.02E-03 | 1.42 | up |
| 3beta,5alpha,6beta-Trihydroxycholestane | 16.78 | 420.3603 | C27H48O3 | 1.83 | 1.62E-02 | 1.53 | up |
| Cinerin II | 11.27 | 360.1937 | C21H28O5 | 1.29 | 2.05E-02 | 1.57 | up |
| N-ethylene glycol neuraminic acid | 1.95 | 325.1009 | C11H19NO10 | 1.64 | 4.37E-03 | 2.13 | up |
| Ginkgolic acid II | 15.19 | 374.2821 | C24H38O3 | 1.00 | 4.07E-02 | -1.30 | down |
| LXA5 | 7.07 | 350.2093 | C20H30O5 | 1.52 | 3.41E-03 | 1.18 | up |
| 4-Chloro-6-{5-[(2-morpholin-4-ylethyl)amino]-1,2-benzisoxazol-3-yl}benzene-1,3-diol | 1.97 | 389.1142 | C19H20ClN3O4 | 1.01 | 7.97E-03 | 1.11 | up |
| [12]-Gingerol | 14.39 | 378.277 | C23H38O4 | 1.70 | 1.51E-02 | 1.07 | up |
| Val-Arg-Glu-Glu | 7.27 | 531.2653 | C21H37N7O9 | 1.55 | 2.25E-02 | 1.13 | up |
| Val-Ala-Ser-Asp | 8.41 | 390.1751 | C15H26N4O8 | 1.48 | 2.84E-02 | 1.64 | up |
| TyrMe-Tyr-OH | 6.72 | 480.1533 | C25H24N2O8 | 1.94 | 7.57E-03 | -5.25 | down |
| TyrMe-HoPhe-OH | 3.55 | 478.174 | C26H26N2O7 | 1.18 | 3.45E-03 | 1.75 | up |
| Stigmastentriol | 17.00 | 446.376 | C29H50O3 | 1.72 | 2.16E-03 | 1.19 | up |
| Ser-Lys-Val-Glu | 6.40 | 461.2486 | C19H35N5O8 | 1.72 | 1.27E-02 | -5.73 | down |
| Rifaximin | 6.92 | 785.3524 | C43H51N3O11 | 1.92 | 7.59E-03 | -3.26 | down |
| Rifamycin W-hemiacetal | 6.81 | 653.2836 | C35H43NO11 | 1.35 | 2.79E-02 | 1.10 | up |
| Pteroside Z | 8.62 | 394.1992 | C21H30O7 | 1.33 | 3.65E-03 | 1.17 | up |
| Polyporusterone D | 15.47 | 460.3189 | C28H44O5 | 1.66 | 3.36E-02 | 1.60 | up |
| N-Succinyl-L-glutamate;(2S)-2-(3-Carboxypropanoylamino)pentanedioic acid | 6.56 | 247.0692 | C9H13NO7 | 1.37 | 1.81E-02 | 1.03 | up |
| N-(4-Methyl-2-oxochromen-7-yl)icosa-5,8,11,14-tetraenamide | 13.14 | 461.293 | C30H39NO3 | 1.66 | 6.61E-03 | -1.53 | down |
| N,N'-(acridine-3,6-diyl)diacetamide | 2.20 | 293.1164 | C17H15N3O2 | 1.62 | 2.31E-02 | 3.17 | up |
| Met-Thr-OH | 3.68 | 358.0835 | C14H18N2O7S | 1.91 | 8.10E-03 | 2.61 | up |
| Maysin 3'-methyl ether | 1.97 | 590.1636 | C28H30O14 | 1.62 | 8.41E-03 | 2.18 | up |
| Lys-HoPhe-OH | 6.25 | 415.1743 | C21H25N3O6 | 1.40 | 4.25E-02 | -1.75 | down |
| Lys-Gln-Ala-Gly-Asp-Val | 8.36 | 616.318 | C25H44N8O10 | 1.54 | 1.17E-02 | 1.11 | up |
| Lucidenic acid A | 11.54 | 458.2668 | C27H38O6 | 1.55 | 4.03E-02 | 1.65 | up |
| Isorhamnetin 3-neohesperidoside | 2.18 | 624.169 | C28H32O16 | 1.13 | 1.39E-02 | 1.00 | up |
| His-Lys-Leu-Val-Val | 15.41 | 594.3853 | C28H50N8O6 | 1.35 | 1.92E-02 | -1.27 | down |
| Hexadecyl ferulate | 15.62 | 418.3083 | C26H42O4 | 1.69 | 6.92E-04 | 1.95 | up |
| Dolichosterone | 15.64 | 462.3345 | C28H46O5 | 1.76 | 2.31E-02 | 2.69 | up |
| Diacetyl-10-gingerdiol | 11.54 | 436.2825 | C25H40O6 | 1.67 | 2.47E-02 | 1.80 | up |
| Demethylphylloquinol | 14.98 | 438.3498 | C30H46O2 | 1.69 | 1.12E-02 | 1.28 | up |
| Asp-Tyr-Ile-Glu | 5.87 | 538.2275 | C24H34N4O10 | 1.46 | 2.40E-02 | -2.74 | down |
| Asp-Ile-Ser-Glu | 5.47 | 462.1962 | C18H30N4O10 | 1.87 | 5.65E-04 | -1.74 | down |
| Asn-Ile | 6.30 | 245.1376 | C10H19N3O4 | 1.67 | 5.03E-03 | 1.40 | up |
| Artonin K | 5.67 | 382.1053 | C21H18O7 | 1.25 | 3.25E-02 | 2.46 | up |
| 9,10,13-Trihydroxystearic acid | 10.17 | 332.2563 | C18H36O5 | 1.51 | 3.76E-03 | -1.23 | down |
| 7(14)-Bisabolene-2,3,10,11-tetrol | 7.32 | 272.1988 | C15H28O4 | 1.93 | 1.70E-03 | 1.29 | up |
| 6k-PGF1d4 | 9.23 | 374.2606 | C20H30D4O6 | 1.57 | 2.02E-02 | 1.04 | up |
| 5,7-dihydroxy-2-(1-hydroxy-3,5-dimethoxy-4-oxocyclohex-2-en-1-yl)-4H-chromen-4-one | 1.72 | 348.0845 | C17H16O8 | 1.66 | 1.07E-03 | 1.88 | up |
| 4-[3-(3,4-dihydroxyphenyl)-2-hydroxypropyl]benzene-1,2,3,5-tetrol | 5.47 | 308.0896 | C15H16O7 | 1.77 | 3.71E-03 | -2.72 | down |
| 3-hydroxydecanoyl carnitine | 10.64 | 331.2359 | C17H33NO5 | 1.82 | 7.77E-04 | -1.18 | down |
| 3-Methyl-5-pentyl-2-furanpentadecanoic acid | 15.88 | 392.329 | C25H44O3 | 1.86 | 7.56E-03 | 1.35 | up |
| 3,4-Methylenesebacic acid | 8.06 | 226.1205 | C12H18O4 | 1.48 | 2.45E-02 | -1.58 | down |
| 2-docosanamidoethanesulfonic acid | 16.43 | 447.3382 | C24H49NO4S | 1.75 | 1.48E-02 | 1.16 | up |
| 2-[(1S,2S,5S)-5-Hydroxy-2-(3-hydroxypropyl)cyclohexyl]-5-(2-methyloctan-2-yl)phenol | 14.00 | 376.2977 | C24H40O3 | 1.71 | 4.30E-02 | 3.49 | up |
| 2-Hydroxy-6-oxo-6-(2-hydroxyphenoxy)-hexa-2,4-dienoate | 9.67 | 250.0477 | C12H10O6 | 1.50 | 2.75E-02 | -1.01 | down |
| 2-(Methoxycarbonyl)-5-methyl-2,4-bis(3-methyl-2-butenyl)-6-(2-methyl-1-oxopropyl)-5-(4-methyl-3-pentenyl)cyclohexanone | 14.02 | 458.3396 | C29H46O4 | 1.37 | 9.81E-03 | 1.71 | up |
| 2,3-dinor, 6-keto-PGF1 | 10.41 | 342.2042 | C18H30O6 | 1.45 | 3.75E-02 | 1.53 | up |
| 19(R)-hydroxy-PGB2 | 7.48 | 350.2093 | C20H30O5 | 1.60 | 3.51E-02 | 1.83 | up |
| 18β-Glycyrrhetinic acid | 15.61 | 470.3396 | C30H46O4 | 1.23 | 2.79E-02 | 1.24 | up |
| 17-beta-Estradiol glucuronide | 6.76 | 448.2097 | C24H32O8 | 1.64 | 9.33E-03 | -1.19 | down |
| 12-epi Leukotriene B4-d4 | 15.25 | 340.2552 | C20H28D4O4 | 1.53 | 7.48E-03 | -1.86 | down |
| 10'-Apo-beta-carotenal | 9.73 | 376.2766 | C27H36O | 2.03 | 2.92E-05 | 1.56 | up |
| (±)7(8)-DiHDPE(A) | 10.44 | 362.2457 | C22H34O4 | 1.77 | 3.87E-02 | 2.67 | up |
| (8E,10S,12Z,15Z)-10-Hydroperoxyoctadeca-8,12,15-trienoate | 16.78 | 310.2144 | C18H30O4 | 1.39 | 3.07E-02 | -1.13 | down |
| (3beta,5alpha,6beta,7alpha,22E,24R)-Ergosta-8,22-diene-3,5,6,7-tetrol | 16.43 | 446.3396 | C28H46O4 | 1.78 | 6.88E-03 | 1.32 | up |
| (3beta,5alpha,6beta,22E,24R)-23-Methylergosta-7,22-diene-3,5,6-triol | 16.46 | 444.3603 | C29H48O3 | 1.79 | 1.31E-02 | 2.34 | up |
| (3beta,17alpha,23S)-17,23-Epoxy-3,29-dihydroxy-27-norlanost-8-en-24-one | 14.73 | 458.3396 | C29H46O4 | 1.99 | 7.52E-03 | 1.95 | up |
| (3beta,17alpha,23R)-17,23-Epoxy-3,29-dihydroxy-27-norlanost-8-ene-15,24-dione | 11.52 | 472.3189 | C29H44O5 | 1.70 | 2.34E-02 | 1.03 | up |
| (3b,21b)-12-Oleanene-3,21,28-triol 28-[arabinosyl-(1->3)-arabinosyl-(1->3)-arabinoside] | 12.57 | 854.5028 | C45H74O15 | 1.53 | 2.26E-02 | 1.16 | up |
| (2Z,8S,9Z)-2,9-Heptadecadiene-8-hydroxy-4,6-diyne-1-yl acetate | 1.90 | 302.1882 | C19H26O3 | 1.51 | 1.85E-02 | 1.33 | up |
| (2S,2'R,3S,3'R,4S)-3,4',5,7-Tetrahydroxyflavan(2->7,4->8)-3,3',5,5',7-pentahydroxyflavan | 14.58 | 560.1319 | C30H24O11 | 1.80 | 7.22E-03 | -2.03 | down |
| (2E)-3-(2,3-dihydroxyphenyl)prop-2-enoic acid | 5.94 | 180.0423 | C9H8O4 | 1.64 | 3.60E-02 | -1.92 | down |
| (23S,24S)-17,23-Epoxy-24,29-dihydroxy-27-norlanost-8-ene-3,15-dione | 12.52 | 472.3189 | C29H44O5 | 1.80 | 4.65E-04 | 1.12 | up |

**Table S2** Short-chain fatty acids precision and recovery rate (n=6)

| NO. | SCFAs | Intra-day precision (Low) RSD% | Intra-day precision (High) RSD% | Daytime precision (Low) RSD% | Daytime precision (High) RSD% | Sample recovery rate (Low) % | Sample recovery rate (Medium) % | Sample recovery rate (High) % |
| --- | --- | --- | --- | --- | --- | --- | --- | --- |
| 1 | AA | 2.85 | 6.07 | 5.95 | 8.38 | 89.80 | 109.29 | 99.57 |
| 2 | PA | 3.11 | 8.08 | 6.41 | 8.71 | 88.78 | 86.73 | 98.23 |
| 3 | IBA | 3.10 | 6.01 | 4.34 | 7.85 | 104.08 | 105.61 | 95.38 |
| 4 | BA | 2.76 | 6.11 | 5.49 | 7.70 | 92.86 | 87.96 | 91.90 |
| 5 | IVA | 3.07 | 7.68 | 5.02 | 7.72 | 93.88 | 96.12 | 90.80 |
| 6 | VA | 2.60 | 5.53 | 4.92 | 7.87 | 87.76 | 95.61 | 87.30 |
| 7 | HA | 2.89 | 7.77 | 5.63 | 7.67 | 104.08 | 100.92 | 85.91 |
